# Unraveling the regulation of sugar beet pulp utilization in the industrially relevant fungus *Aspergillus niger*

**DOI:** 10.1016/j.isci.2022.104065

**Published:** 2022-03-12

**Authors:** Sandra Garrigues, Roland S. Kun, Mao Peng, Diane Bauer, Keykhosrow Keymanesh, Anna Lipzen, Vivian Ng, Igor V. Grigoriev, Ronald P. de Vries

**Affiliations:** 1 Fungal Physiology, Westerdijk Fungal Biodiversity Institute & Fungal Molecular Physiology, Utrecht University, Uppsalalaan 8, 3584 CT Utrecht, the Netherlands; 2 USA Department of Energy Joint Genome Institute, Lawrence Berkeley National Laboratory, 1 Cyclotron Road, Berkeley, CA 94720, USA

**Keywords:** Microbial metabolism, Mycology

## Abstract

Efficient utilization of agro-industrial waste, such as sugar beet pulp, is crucial for the bio-based economy. The fungus *Aspergillus niger* possesses a wide array of enzymes that degrade complex plant biomass substrates, and several regulators have been reported to play a role in their production. The role of the regulators GaaR, AraR, and RhaR in sugar beet pectin degradation has previously been reported. However, genetic regulation of the degradation of sugar beet pulp has not been assessed in detail. In this study, we generated a set of single and combinatorial deletion mutants targeting the pectinolytic regulators GaaR, AraR, RhaR, and GalX as well as the (hemi-)cellulolytic regulators XlnR and ClrB to address their relative contribution to the utilization of sugar beet pulp. We show that *A. niger* has a flexible regulatory network, adapting to the utilization of (hemi-)cellulose at early timepoints when pectin degradation is impaired.

## Highlights

• Major sugar beet pulp components are sequentially utilized by *A. niger*
• Contribution of major regulators toward sugar beet pulp utilization was compared
• Deletion of *araR* and *clrB* showed high impact on growth after 8 and 24 h, respectively

## Graphical abstract

**Figure:**
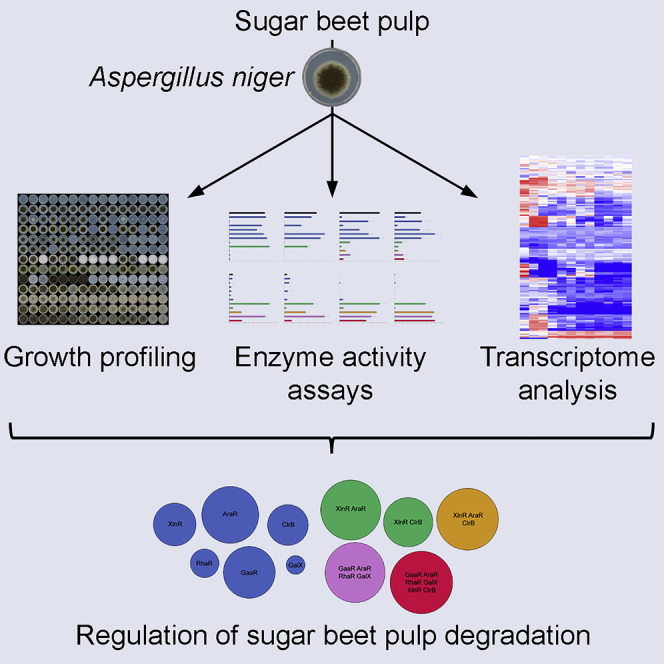

## Introduction

Plant biomass is the most abundant renewable terrestrial resource, and is considered a valuable raw material for an increasing number of biotechnological applications such as pulp and paper, food and feed, textiles, detergents, biofuels, and biochemicals (de Vries and Visser, 2001). It mainly consists of plant cell wall polysaccharides (cellulose, hemicellulose, pectin), lignin, proteins, and storage polysaccharides (starch, inulin, and gums) (de Vries and Mäkelä, 2020). Achieving efficient use of plant biomass as feedstock is crucial in the current bio-based economy scenario of valorizing renewable resources. In this context, low-cost plant biomass substrates are of high interest.

Sugar beet pulp is the main by-product of industrial sugar beet (*Beta vulgaris*) processing, and is currently sold as low-value animal feed. Around 30 million tons of sugar beets are produced annually in the USA only, generating over 1.5 million tons of sugar beet pulp as dry residue (Zhang et al., 2020). It has been reported that 1 ton of sugar beet yields approximately 150 kg of sugar and 500 kg of wet beet pulp (or 210 kg pressed beet pulp, or 50 kg dehydrated beet pulp) (Legrand, 2005). The degradation of sugar beet pulp polymeric carbohydrates into monosaccharides is a promising step toward increasing the value of this by-product of the sugar industry. This substrate is especially rich in cellulose (20%–24%), hemicellulose (25%–36%) (mainly xyloglucan), and pectin (15%–25%) (Thibault and Rouau, 1990), of which the structure has been well studied (de Vries and Visser, 2001).

Filamentous fungi, particularly ascomycetes and basidiomycetes, are highly efficient degraders of plant biomass (Bomble et al., 2017; Mäkelä et al., 2014). They secrete large amounts or hydrolytic and oxidative enzymes to efficiently degrade the complex structure of plant material (de Vries et al., 2020; van den Brink and de Vries, 2011). These enzymes have been catalogued in the Carbohydrate Active enZyme (CAZy) database (www.cazy.org) in several families and subfamilies according to amino acid sequence similarity and enzymatic activities (Lombard et al., 2014). Filamentous fungi control the production of plant polysaccharide-degrading enzymes at the transcriptional level to ensure a space-time balanced and optimized enzyme production. Transcription factors (TFs) are regulatory proteins that activate or repress gene expression by specific binding to conserved motifs in the promoters of their target genes. Several TFs involved in the regulation of plant biomass utilization have been characterized in fungi (Benocci et al., 2017).

*Aspergillus niger* is a biotechnologically relevant ascomycete with a long history of safe use for the production of enzymes and metabolites (Cairns et al., 2018; Frisvad et al., 2018). This fungus has a great potential for plant biomass degradation (de Vries et al., 2017; Kun et al., 2021; Meijer et al., 2011), and is the most commonly used species in industry. In *A. niger,* sugar-specific TFs are activated or repressed by the presence of monomeric sugars or intracellular compounds thereof. The TFs involved in the degradation of the polysaccharides present in sugar beet pulp (cellulose, xyloglucan, and pectin) and the utilization of the resulting monosaccharides are of particular interest for sugar beet pulp valorization. Three transcriptional activators, namely the galacturonic acid-responsive regulator GaaR, the rhamnose-responsive regulator RhaR, and the arabinanolytic regulator AraR, have already been described in *A. niger* (Alazi et al., 2016; Battaglia et al., 2011; Gruben et al., 2014), and their co-regulation for pectin degradation has been studied in this fungus (Kowalczyk et al., 2017), with GaaR playing the most dominant role. D-galactose is particularly present in xyloglucan, pectin, and gums (mainly galacto(gluco)mannans) (Kowalczyk et al., 2014). In *A. niger,* the galactose-responsive regulator GalX has been reported to play a key role in D-galactose catabolism via the oxido-reductive pathway (Gruben et al., 2012). Moreover, four other TFs have been reported to be involved in (hemi-)cellulose degradation in this species: the xylanolytic regulator XlnR (Van Peij et al., 1998b); AraR, which not only controls pectin and hemicellulose degradation in *A. niger* (de Souza et al., 2013; Kowalczyk et al., 2017) but also the Pentose Catabolic Pathway (PCP) together with XlnR (Battaglia et al., 2014); and the cellulose-responsive regulators ClrA and ClrB (Raulo et al., 2016). However, ClrB plays a more dominant role in the process of cellulose degradation than ClrA in *A. niger* (Kun et al., 2021; Raulo et al., 2016), and both have been suggested to be under the control of XlnR (Raulo et al., 2016).

The present work aims to study the relative contribution of a set of TFs – GaaR, AraR, RhaR, GalX, XlnR, and ClrB – to the degradation of sugar beet pulp by *A. niger*. For this purpose, we generated a combination of single and multiple deletion mutant strains in these six regulators, and their phenotype and genetic response on sugar beet pulp were analyzed.

## Results

### Combined deletion of GaaR, AraR, RhaR, GalX, XlnR, and ClrB abolished the growth of *A. niger* on sugar beet pulp

**Figure 1:**
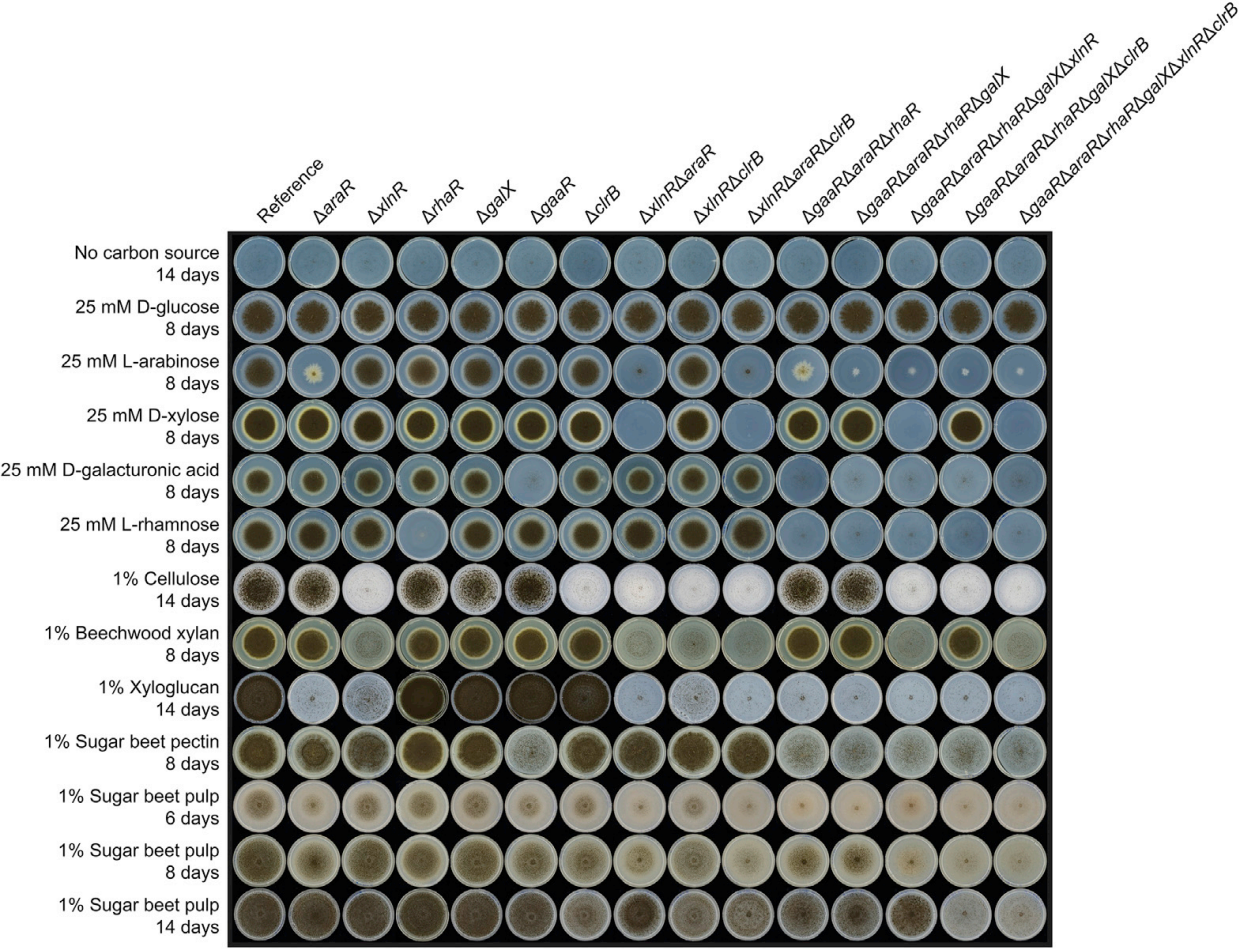
Phenotypic analysis of *A. niger* reference (CBS 138852) and regulatory mutant strains grown on sugar beet pulp and related carbon sources for up to 14 days at 30°C

In order to study whether the six TFs chosen in this work (GaaR, AraR, RhaR, GalX, XlnR, and ClrB) affect *A. niger* growth on sugar beet pulp, single and multiple deletion mutants were obtained and their phenotypes on washed sugar beet pulp and related carbon sources were analyzed (Figure 1). The growth of D-glucose was used as an internal control and was similar in all strains. The Δ*clrB* mutant and all mutant combinations containing *clrB* deletion highly reduced *A. niger* growth on sugar beet pulp. Δ*araR,* Δ*gaaR,* and, to a lesser degree, Δ*rhaR* mutants showed a slight reduction of growth on sugar beet pulp after 8 days of growth, whereas the growth of Δ*xlnR* and Δ*galX* was comparable with that of the parental strain. Interestingly, Δ*araR,* Δ*xlnR,* Δ*rhaR,* Δ*galX*, and Δ*gaaR* showed a similar growth pattern on sugar beet pulp compared with the reference after 14 days of growth. These results suggest that among the six TFs used in this study, ClrB is the most dominant regulator for sugar beet pulp degradation in *A. niger* solid cultures, likely owing to the high cellulose content of this crude substrate. Surprisingly, Δ*gaaR* did not show a differential phenotype on sugar beet pulp compared with that of the reference strain at late timepoints, even though GaaR is the most dominant regulator of pectin, which is one of the main components of sugar beet pulp. In contrast, the pectin-related mutants Δ*gaaR*Δ*araR*Δ*rhaR* and Δ*gaaR*Δ*araR*Δ*rhaR*Δ*galX* showed reduced growth ability on sugar beet pulp. Finally, the sextuple mutant Δ*gaaR*Δ*araR*Δ*rhaR*Δ*galX*Δ*xlnR*Δ*clrB* almost completely abolished *A. niger*’s ability to grow on sugar beet pulp and all tested sugar beet pulp-related carbon sources (except for D-glucose) at all timepoints. In order to reveal the individual contribution of the different regulators on sugar beet pulp degradation, growth was also evaluated on the polymeric and monomeric components of this crude substrate (Figure 1). No growth was observed for Δ*xlnR* and Δ*clrB* on cellulose, confirming the key role of XlnR and ClrB in cellulose degradation. In addition, Δ*araR,* followed by Δ*xlnR,* showed a strongly reduced growth on xyloglucan, the most abundant hemicellulose present in sugar beet pulp. As expected, Δ*gaaR* showed poor growth on pectin and D-galacturonic acid. Despite D-galactose being an abundant component of pectin, *galX* deletion did not significantly affect the growth of *A. niger* on pectin, although its deletion strongly affected the growth on L-arabinose in multiple combinatorial deletion mutants (Figure 1). This would suggest a possible involvement of GalX in L-arabinose utilization in this fungus, which is also a major pectin component.

Taking all these results together, abolished growth on sugar beet pulp and all sugar beet pulp components can only be accomplished with the combinatorial deletion of all TFs of this study, suggesting that they all play a role in sugar beet pulp utilization.

### AraR plays a major role in the regulation of extracellular activities in sugar beet pulp liquid cultures

To study the possible correlation between reduced growth ability on sugar beet pulp shown by the different mutant strains (Figure 1) and protein production levels, fungal mycelia were transferred to liquid medium containing 1% washed sugar beet pulp, and cell-free supernatant samples of selected strains grown for 2, 8, and 24 h were analyzed by SDS-PAGE (Figure S1). No protein production could be detected in any of the supernatants after 2 h of incubation on sugar beet pulp (data not shown). Deletion of *araR* or *gaaR* had the highest impact on the overall extracellular proteins produced, which was evident after 8 h of growth. This pattern positively correlates with the growth reduction of Δ*araR* and Δ*gaaR* on sugar beet pulp shown at early timepoints (Figure 1), which disappears after 14 days of growth. In contrast, deletion of *clrB* did not cause a significant change in the protein production pattern compared with that of the reference strain, despite the high impact of *clrB* deletion on growth on sugar beet pulp (Figure 1). All combinatorial mutants showed a highly reduced ability to produce extracellular proteins, with the quadruple Δ*gaaR*Δ*araR*Δ*rhaR*Δ*galX* and the sextuple Δ*gaaR*Δ*araR*Δ*rhaR*Δ*galX*Δ*xlnR*Δ*clrB* mutants showing the same protein production pattern. However, the aforementioned quadruple mutant still showed residual growth ability on sugar beet pulp, whereas the growth of the sextuple mutant was abolished (Figure 1).

**Figure 2:**
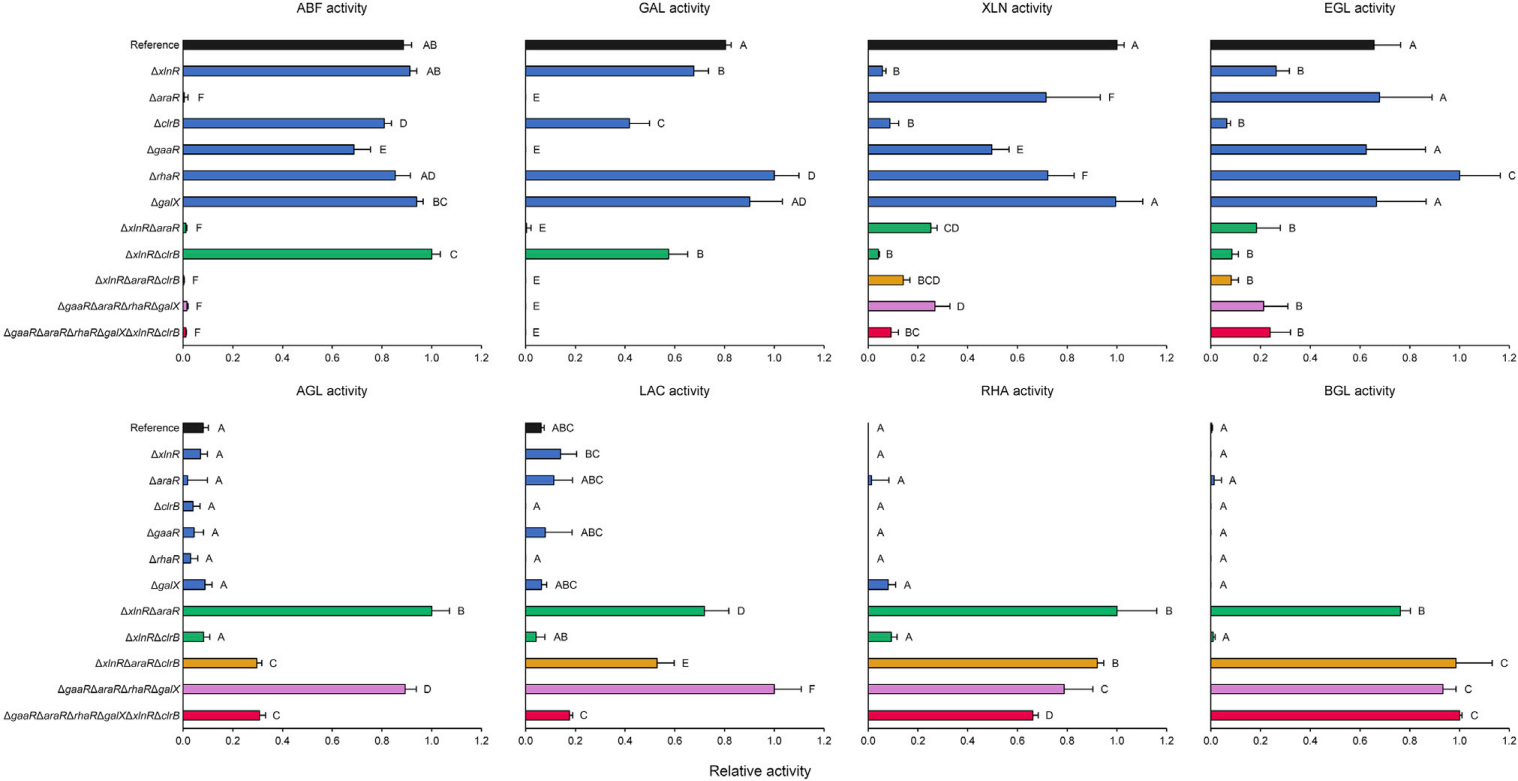
Enzyme activity assays of supernatants from *A. niger* (CBS 138852) reference and deletion mutant strains The reference, and the selected single-, double-, triple-, quadruple-, and sextuple deletion mutant strains are indicated by different colors. Data represent the normalized mean activity values of biological duplicates and technical triplicates and the standard deviation. The raw absorbance values measured at 405 or 600 nm wavelength and the calculated activity values are indicated in Data S1. ABF, α-L-arabinofuranosidase; GAL, endo-1,4-β-galactanase; XLN, endo-1,4-β-xylanase; EGL, endo-1,4-β-glucanase; AGL, α-1,4-D-galactosidase; LAC, β-1,4-D-galactosidase; RHA, α-L-rhamnosidase; BGL, β-1,4-D-glucosidase activity. Letters (A–F) are shown to explain the statistical differences between samples within each specific enzyme assay. Samples annotated with different letters show significant differences among the strains, whereas samples sharing the same letters show no statistically significant differences (ANOVA and Tukey's HDS test, *p* <0.05).

Assays were performed to evaluate the enzymatic activities present in the exoproteome of the reference and deletion mutant strains (Figure 2). The deletion of *xlnR* and *clrB* significantly reduced endoxylanase (XLN) and endoglucanase (EGL) activities, which was also observed for Δ*xlnR*Δ*clrB*. Moreover, the deletion of *clrB* had a higher impact on the endogalactanase (GAL), arabinofuranosidase (ABF), and β-galactosidase (LAC) activities than that of *xlnR*. These activities contribute to the degradation of pectin, which is abundantly present in sugar beet pulp. However, the growth profile results do not indicate a major involvement of ClrB in the degradation of sugar beet pectin (Figure 1). The deletion of *rhaR* or *galX* resulted in overall little or no change in the measured activities compared with the reference strain. In contrast, the deletion of *araR* or *gaaR* abolished GAL activity, whereas the Δ*araR* strain also showed abolished ABF activity. All combinatorial deletion mutants carrying the deletion of *araR* (Δ*xlnR*Δ*araR*, Δ*xlnR*Δ*araR*Δ*clrB*, Δ*gaaR*Δ*araR*Δ*rhaR*Δ*galX*, and Δ*gaaR*Δ*araR* Δ*rhaR*Δ*galX*Δ*xlnR*Δ*clrB*) showed abolished ABF and GAL activities, as well as highly reduced XLN and EGL activities. Interestingly, these strains also showed significantly increased α-galactosidase (AGL), LAC, α-rhamnosidase (RHA), and β-glucosidase (BGL) activities. These activities were minimal in the exoproteome of the reference, Δ*xlnR*Δ*clrB*, and the single deletion strains. These results may indicate a shift toward the utilization of alternative components of sugar beet pulp by the combinatorial deletion mutants carrying the deletion of *araR*.

### Gene expression levels show the preferential use of sugar beet pulp components by *A. niger*

**Figure 3:**
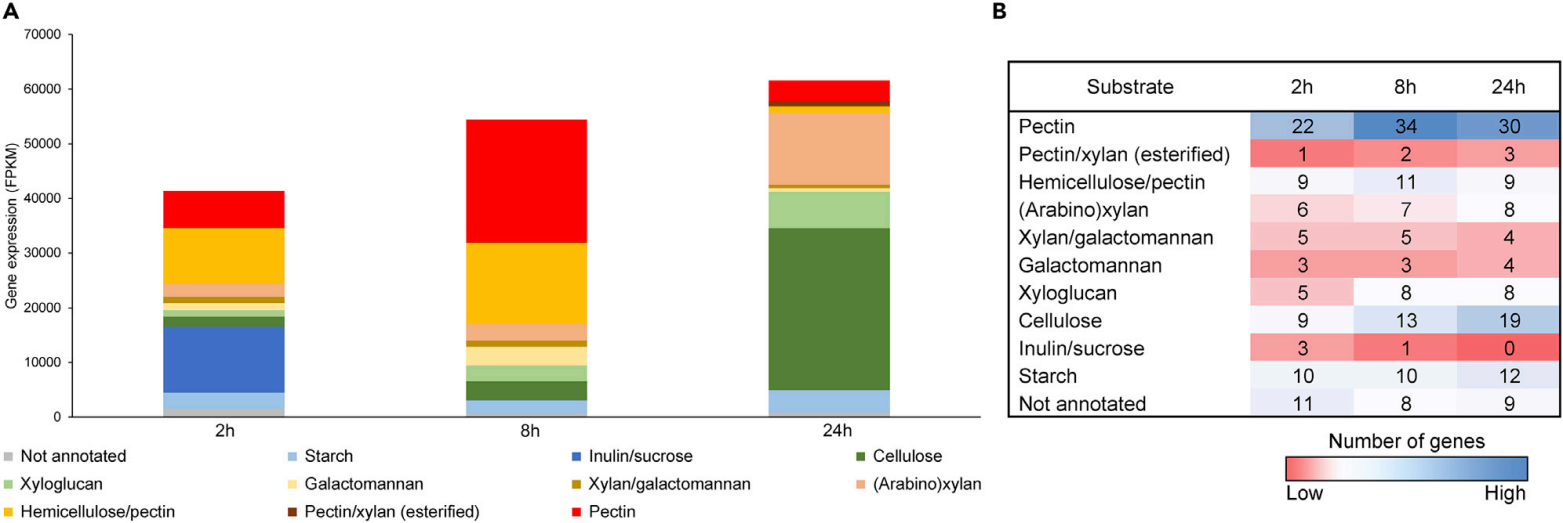
Expression of CAZy-encoding genes in the reference strain (CBS 138852) (A) Cumulative expression of genes associated with the degradation of specific substrates after 2, 8, and 24 h of growth in 1% sugar beet pulp liquid cultures. Substrates are shown in the figure legend. (B) Number of genes associated with the degradation of each substrate at individual timepoints. Only genes with an expression value of FPKM >20 were considered for this analysis.

Transcriptome analysis of 2, 8, and 24 h liquid culture samples has been performed to assess the genetic response of *A. niger* toward the utilization of sugar beet pulp. The expression profile of CAZy-encoding genes of this fungus showed a significant change over time, indicating the preferential use of sugar beet pulp components at different timepoints and the adaptation of *A. niger* to the remaining carbon sources over time (Figure 3).

**Figure 4:**
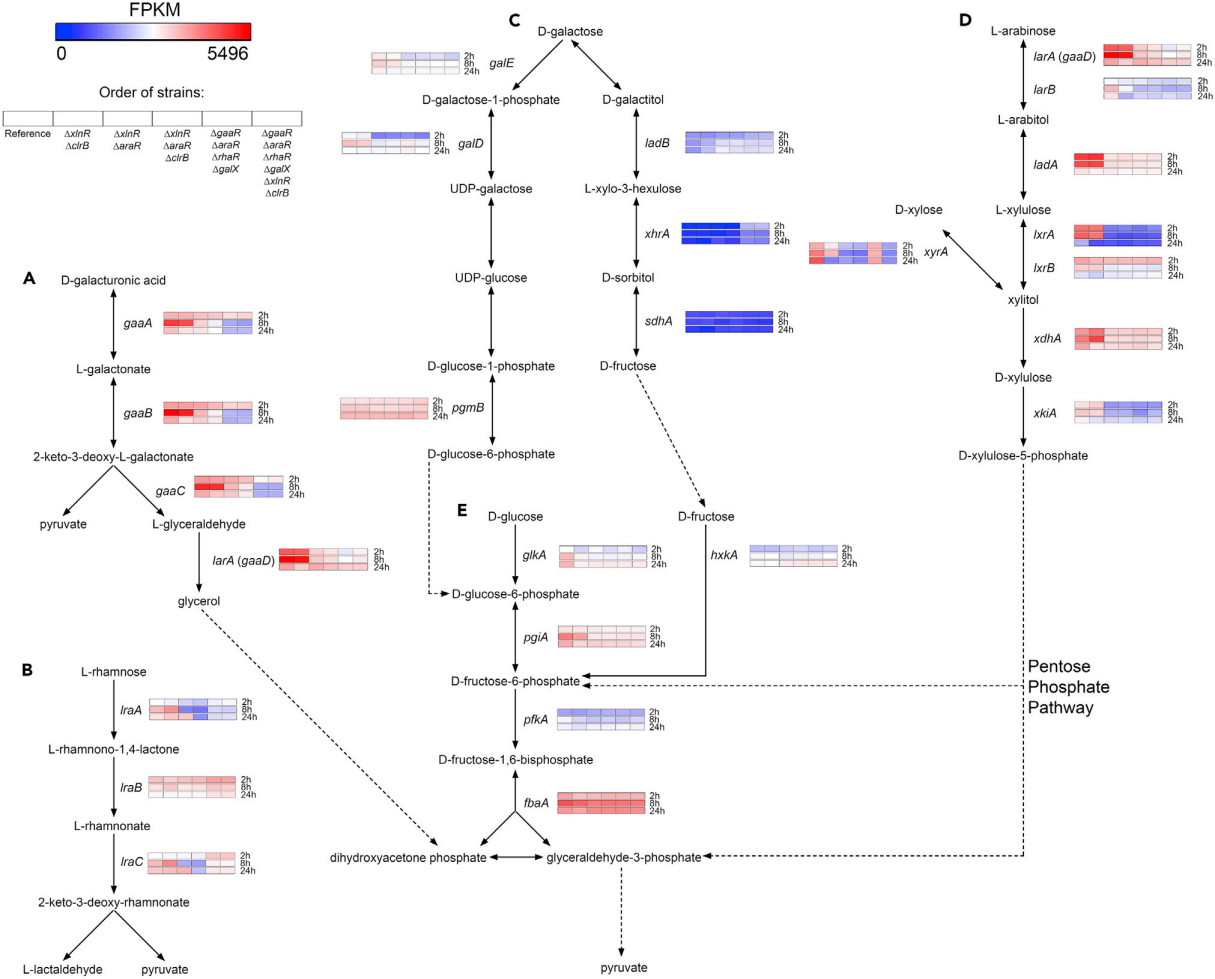
Hierarchical clustering of genes involved in the major primary carbon metabolic pathways in the *A. niger* reference (CBS 138852) and selected combinatorial deletion strains Gene expression data are presented for the D-galacturonic acid pathway (A), L-rhamnose pathway (B), the Leloir and oxidoreductive D-galactose pathway (C), Pentose Catabolic Pathway (D), and glycolysis (E). Dashed lines indicate the connections between different pathways when applicable.

The exo-inulinase-encoding gene *inuE* showed the highest expression level after 2 h (Data S2), most likely as a response to the presence of sucrose that may still be present in washed sugar beet pulp. Moreover, *sucA*, encoding an extracellular invertase, was among the highest expressed CAZy genes at this timepoint. The high expression level of these two enzymes at this early timepoint, as well as the minimal/abolished expression after 8 and 24 h of growth indicates that *A. niger* first utilizes sucrose as a means of quick recovery after the transfer to sugar beet pulp. Several pectinolytic and/or hemicellulolytic genes (e.g., *abfB*, *abnA*, *abfC*, *axhA*, NRRL3_8701 (putative exo-galactanase encoding gene), *gbgA*, *lacA*, *abfA*, *lacB*, *eglA*, *galA*) also showed high expression levels (FPKM >500) in the reference strain after 2 h of growth. Expression analysis of genes involved in the primary sugar metabolic pathways (D-galacturonic acid pathway (Figure 4A); L-rhamnose pathway (Figure 4B); D-galactose pathways (Figure 4C); pentose catabolic pathway (PCP) (Figure 4D); and glycolysis (Figure 4E)) showed that the expression of the PCP genes was the highest at this stage of growth (Figure 4D). This result correlates with the expected high release of L-arabinose catalyzed by the aforementioned CAZymes.

The relative expression of pectinolytic and hemicellulolytic (including (arabino)xylanases, galactomannanases, xyloglucanases) genes was higher after 8 h (Figure 3). The genes encoding pectinolytic enzymes showed the highest increment in expression (Data S2), indicating that *A. niger* mainly utilizes pectin at this timepoint. This is further supported by the increased expression of genes involved in D-galacturonic acid metabolism (*gaaA, gaaB*, *gaaC*, and *larA*) (Figure 4A), which is the main constituent of the pectin backbone.

The expression level of cellulolytic genes was relatively low after 2 or 8 h, which correlates with our observation that cellulose is utilized only at a later stage of growth (Figure 1). However, the significant decrease in the expression of pectinolytic genes, as well as the increase in case of cellulolytic genes (e.g., *cbhB*, *cbhC*, *eglA*, *bgl4*, *bglA*, *eglC*, *bglM*, NRRL3_3383 (putative lytic polysaccharide monooxygenase (LPMO)) and NRRL3_6436 (putative β-glucosidase) (Data S2) after 24 h, indicates a shift toward cellulose utilization at this timepoint (Figure 3).

Several genes coding for enzymes involved in starch degradation (e.g., *agdB*, *glaA*, *amyA*, *agdA*, *aamA*) (Data S2) also showed an overall consistent expression at each timepoint. However, considering the composition of sugar beet pulp, it was not likely that the fungus utilized starch for growth at any of the studied timepoints. It is most likely that the presence of low levels of D-glucose in the medium induced the production of the aforementioned starch-degrading enzymes mediated by the amylolytic regulator AmyR (Petersen et al., 1999; vanKuyk et al., 2012).

### The contribution of major TFs toward the degradation of sugar beet pulp is time-dependent

Among the strains of this study, only the strains carrying the deletion of *araR* (Δ*araR,* Δ*xlnR*Δ*araR*, Δ*xlnR*Δ*araR*Δ*clrB*, Δ*gaaR*Δ*araR*Δ*rhaR*Δ*galX*, Δ*gaaR*Δ*araR*Δ*rhaR*Δ*galX*Δ*xlnR*Δ*clrB*) showed a different CAZyme pattern from the reference strain after 2 h of growth (Figure S2, clusters F and G). However, the reduced or impaired pectinolytic and hemicellulolytic activities in these deletion mutants did not result in the upregulation of genes involved in the degradation of other components, such as cellulose, at this timepoint. Moreover, none of the single or combinatorial TF deletions resulted in a significant change (fold change >2 or <0.5, and *p*adj <0.01) in the expression level of *inuE* and *sucA* (Figure S2). These results support the primary utilization of sucrose by each deletion mutant after 2 h of growth.

**Figure 5:**
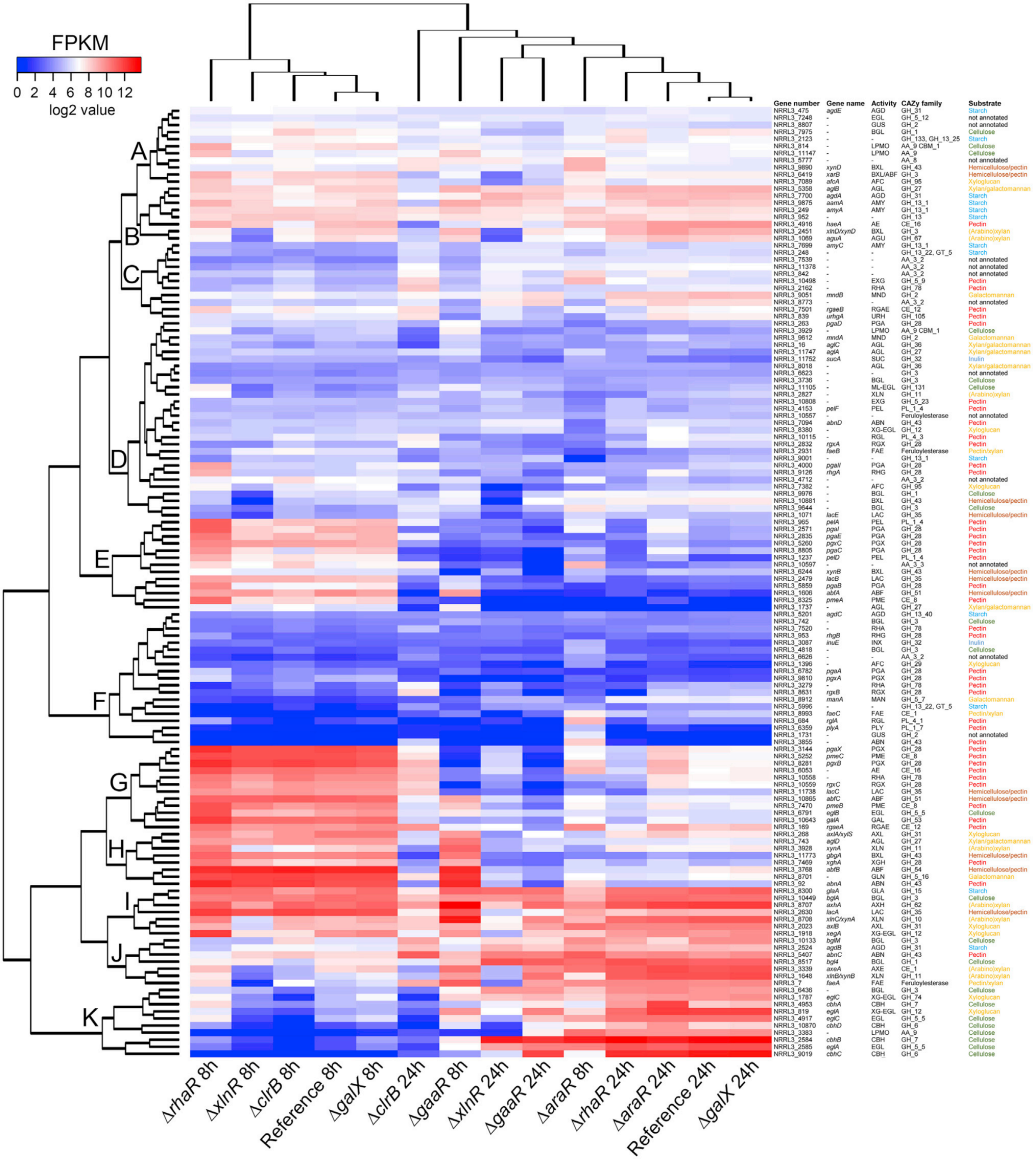
Hierarchical clustering of CAZy-encoding genes in the *A. niger* reference (CBS 138852) and single deletion mutants Gene expression data originated from 8 to 24 h of growth in 1% sugar beet pulp liquid cultures. The substrates associated with the corresponding genes are indicated by different colors. Enzyme activity abbreviations are described in Table S2.

**Figure 6:**
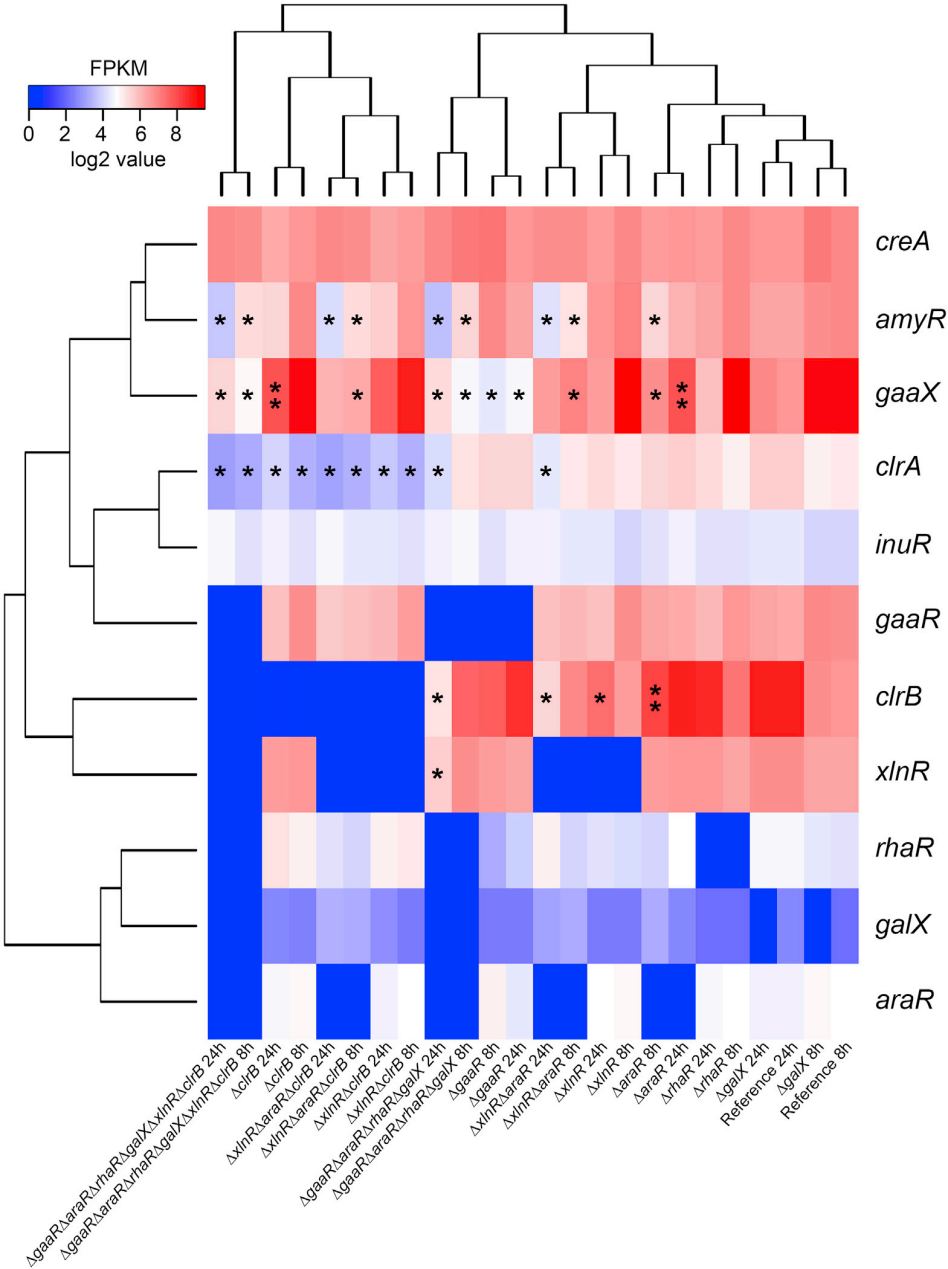
Hierarchical clustering of transcription factor genes in the *A. niger* reference (CBS 138852) and deletion mutant strains Gene expression data originated from 8 to 24 h of growth in 1% sugar beet pulp liquid cultures. Genes that are downregulated compared with the reference (fold change <0.5; *p*adj <0.01) are indicated by an asterisk (∗). Genes that are upregulated compared with the reference (fold change >2; *p*adj <0.01) are indicated by two asterisks (∗∗).

In contrast, after 8 and 24 h, several single and combinatorial deletion mutants indicated a substantial alteration in the utilization of sugar beet pulp components. Hierarchical clustering of the expression of CAZy-encoding genes after 8 h highlighted the important role of AraR and GaaR in the degradation of sugar beet pulp (Figure 5). Both single deletions resulted in the downregulation of a wide range of pectinolytic genes (Figure 5, cluster G). Moreover, the deletion of *araR* resulted in the downregulation of additional genes encoding accessory enzymes required for the efficient degradation of pectin (*gbgA*, *abfB*, *lacA*) (Figure 5, clusters H and I). The gene encoding the repressor of D-galacturonic acid utilization, GaaX (Niu et al., 2017) showed high expression in the reference strain after 8 h of growth, when the fungus was most likely utilizing pectin as primary carbon source. Both the single and combinatorial *araR* and *gaaR* deletion mutants showed the downregulation of *gaaX* after 8 h compared with the reference strain (Figure 6). However, only the mutants carrying the deletion of *gaaR* showed downregulation of *gaaX* after 24 h of growth (Figure 6).

Several genes were also upregulated in Δ*araR* and/or Δ*gaaR*. Several cellulolytic genes (e.g., *cbhB*, *eglA*, *eglC*, and NRRL3_6436 (putative β-glucosidase encoding gene)) (Figure 5, cluster K) were highly expressed when either *araR* or *gaaR* was deleted. Moreover, the β-glucosidase encoding genes *bglM* and *bgl4*, the cellobiohydrolase encoding genes *cbhA* and *cbhC*, as well as the putative LPMO encoding gene NRRL3_3383 were also upregulated in the *araR* deletion mutant (Figure 5, clusters J and K). The majority of these cellulolytic genes (*cbhB*, *cbhC*, *cbhD*, *eglA*, NRRL3_3383) showed minimal expression in the reference strain, which correlates with the increased number of cellulolytic genes expressed in the Δ*araR* and Δ*gaaR* mutants (Figure S3). Moreover, the increased cellulolytic gene expression also correlates with the higher expression of *clrB* in Δ*araR* (Figure 6). These results suggest an early switch toward the utilization of cellulose in the studied deletion mutants. Moreover, a possible shift toward the utilization of hemicellulose components has been observed in these two single deletion mutants. Hemicellulose-specific upregulated genes include *axeA*, *xlnB/xynB*, *faeA*, *eglA*, and *eglC* (Figure 5, clusters J and K). Overall, the deletion of *gaaR* further increased the expression of these genes compared with the deletion of *araR*, and resulted in the upregulation of additional (arabino)xylanase encoding genes, such as *xynA*, *axhA*, and *xlnC*/*xynA* (Figure 5, clusters H and I; Figure S3).

After 24 h, the reference strain showed the high expression of several (hemi-)cellulases (Figure 5, clusters J and K), indicating mainly the utilization of cellulose and xyloglucan at this stage of growth. Based on the hierarchical clustering of CAZy-encoding genes, the *xlnR* and *clrB* single deletion mutants showed a distinct pattern from that of the reference strain. In Δ*xlnR*, a broad range of CAZy genes encoding several (hemi-)cellulases (Figure 5, clusters H–K), as well as several pectinolytic enzymes, such as *pgaX*, *pmeC*, or *pgxB* (Figure 5, cluster G) were downregulated. Although the deletion of *xlnR* did not result in the upregulation of any CAZy-encoding genes, several cellulolytic genes, such as *cbhB* and *bgl4*, were the most highly expressed CAZyme genes in this mutant (Figure 5, clusters J and K; Figure S4). This result may suggest an attempt of the fungus to use the cellulose present in sugar beet pulp, although the growth profile showed the inability of Δ*xlnR* to grow on this component (Figure 1).

In contrast, the deletion of *clrB* resulted in both the downregulation and upregulation of several CAZy-encoding genes, mainly affecting cellulose utilization (Figure 5, clusters J and K). Moreover, the expression of the cellulolytic regulator encoding gene *clrA* was reduced in the *clrB* deletion mutant (Figure 6), which may explain (part of) the reduction of the expression of cellulolytic genes. Moreover, a large number of hemicellulolytic genes were downregulated in this mutant (Figure 5, clusters H–K), mainly affecting the utilization of xyloglucan. However, several genes involved in pectin utilization were upregulated compared with the reference strain (Figure 5, cluster G; Figure S4), indicating that in contrast to the reference strain, the *clrB* deletion mutant did not shift to the utilization of cellulose after 24 h, and continuously utilized the residual pectin found in sugar beet pulp for growth.

Based on the hierarchical clustering of CAZyme genes at both 8 and 24 h, the deletion of *galX* did not result in a differential expression of any of the genes analyzed.

### Combining TF deletions forces *A. niger* to switch to the utilization of alternative carbon sources

**Figure 7:**
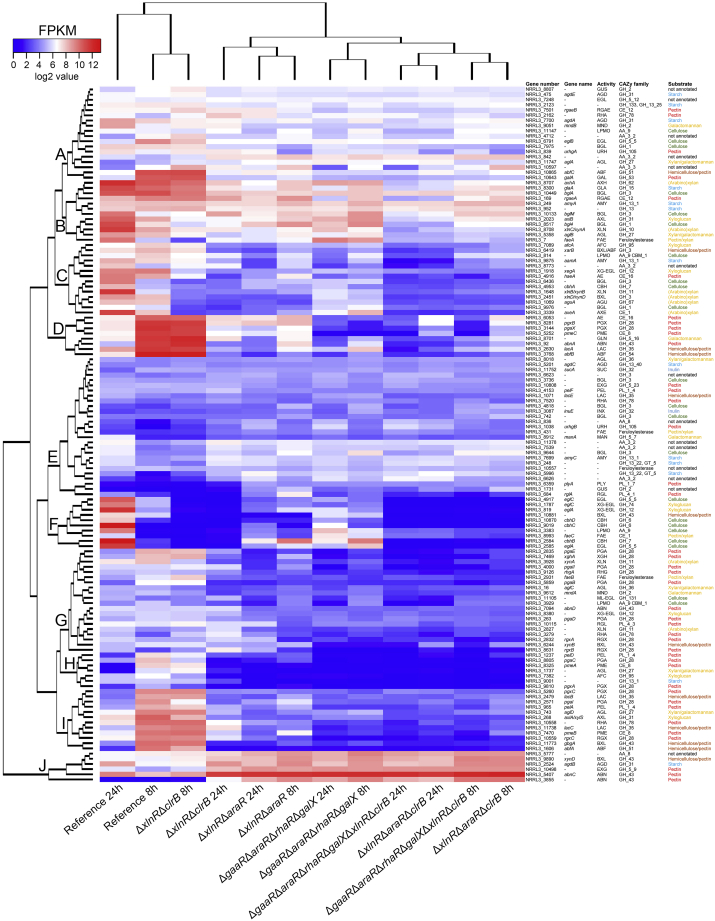
Hierarchical clustering of CAZy-encoding genes in the *A. niger* reference (CBS 138852) and combinatorial deletion mutants Gene expression data originated from 8 to 24 h of growth in 1% sugar beet pulp liquid cultures. The substrates associated with the corresponding genes are indicated by different colors. Enzyme activity abbreviations are described in Table S2.

Transcriptome analysis of combinatorial deletion mutants was assessed to evaluate the interactions between TFs within the regulatory network governing sugar beet pulp degradation. The Δ*xlnR*Δ*clrB* double mutant was primarily expected to impact cellulose and xyloglucan utilization. This mutant did not show a distinct CAZyme profile from that of the reference strain after 8 h, as it did not have a significant impact on the pectinolytic system. Thus, its CAZyme gene expression profile mostly resembles that of the reference strain. However, after 24 h, the Δ*xlnR*Δ*clrB* mutant showed a strong downregulation of major (hemi-)cellulolytic genes (Figure 7, see clusters B, C, and F as examples). Most pectinolytic genes were not affected in this mutant, suggesting that it mainly utilizes residual pectin for growth, similar to Δ*clrB* after 24 h (Figure S4).

All combinatorial deletion mutants, except for Δ*xlnR*Δ*clrB*, showed a strong downregulation of the major pectinolytic genes (Figure 7, clusters D, G, H, and I) after 8 h. However, several (putative) pectinolytic genes (*abnC*, NRRL3_10,498 (putative exo-galactanase encoding gene), and NRRL3_3855 (putative endo-arabinanase encoding gene)) (Figure 7, cluster J) were highly upregulated in most combinatorial deletion strains after both 8 and 24 h. Whereas most combinatorial deletion mutants showed the upregulation of these pectinolytic genes, the quadruple deletion strain Δ*gaaR*Δ*araR*Δ*rhaR*Δ*galX* also showed the upregulation of several cellulolytic genes (*bglM*, *blg4*, NRRL3_9644 (putative β-glucosidase encoding gene), NRRL3_3383 (putative LPMO encoding gene), *cbhB*, and *eglA*) (Figure 7, clusters B, E, and F). All these genes, except for *bglM* and *bgl4*, showed minimal to no expression in the reference strain, indicating that the quadruple deletion mutant may have switched to the utilization of cellulose after 8 h of growth, similar to Δ*araR* and Δ*gaaR* (Figures 5 and S3).

Moreover, all strains, including the reference, showed expression of proteolytic genes after 8 h of growth (Figure S5), which was higher in mutants carrying the *araR* deletion. The aspartic peptidase gene *pepA* showed the highest increase in the expression in the *araR* deletion mutants. The expression of proteolytic genes was, in general, lower at 24 h, with the exception of Δ*xlnR*Δ*clrB*, most likely as an alternative for the inability to utilize cellulose at this timepoint. In contrast, the Δ*xlnR*Δ*araR*Δ*clrB* mutant showed a low expression of selected proteolytic genes (Figure S5), as well as an overall strongly downregulated CAZyme gene profile comparable with that of the Δ*gaaR*Δ*araR*Δ*rhaR*Δ*galX*Δ*xlnR*Δ*clrB* mutant (Figure 7). This indicates that both strains are most likely unable to efficiently utilize any of the sugar beet pulp components after 24 h of growth.

## Discussion

In the bio-based economy, it is crucial to understand the fungal regulatory network governing plant biomass degradation to facilitate the generation of fungal strains with improved abilities to degrade major plant biomass substrates. In this study, we assessed the regulatory network of *A. niger* involved in the degradation of a bulk agro-industrial waste material, sugar beet pulp. We generated a broad set of single and combinatorial deletion mutants targeting the pectinolytic regulators GaaR, AraR, RhaR, and GalX as well as the (hemi-)cellulolytic regulators XlnR and ClrB to evaluate the degradation of major sugar beet pulp components.

Sugar beet pulp mainly consists of pectin, cellulose, and xyloglucan (Thibault and Rouau, 1990), as confirmed by the sugar composition analysis (Table S1). The process of sugar beet pulp degradation by *A. niger* in both solid and liquid cultures has already been reported (Benoit et al., 2015; Garrigues et al., 2021). Moreover, the contribution of three major TFs to the degradation of sugar beet pectin was previously assessed (Kowalczyk et al., 2017). However, the contribution of the major TFs to the overall degradation of sugar beet pulp by *A. niger* has not been reported in detail. Transcriptome data of the *A. niger* reference (CBS 138852) strain showed the preferential use of sugar beet pulp components of this fungus, which correlates with previously reported observations (Garrigues et al., 2021). Our data confirmed that after 2 h, sucrose was the primary carbohydrate of sugar beet pulp utilized by *A. niger*. The gene showing the overall highest expression level in all strains at this timepoint was *inuE*, encoding an exo-inulinase that has been reported to be highly expressed in the presence of sucrose (Yuan et al., 2006). However, the expression of metabolic genes also suggests an early utilization of L-arabinose residues, indicated by the expression of major PCP genes involved in its metabolism. Thus, the response to the presence of sucrose is closely followed by the degradation of pectin, which is most likely followed by the degradation of hemicellulose (e.g., xyloglucan), supported by the high expression level of genes related to degradation of these substrates after 8 and 24 h of growth. Cellulose serves as a last resort substrate, mainly evidenced by the 24 h transcriptome data. The response to the presence of cellulose is most likely triggered when the pectin and hemicellulose content is depleting. The slow utilization of cellulose by *A. niger* was already described (Garrigues et al., 2021; Kun et al., 2021), and is further supported by our growth profile results, showing growth only after long incubation times.

Among all single deletion mutants, the Δ*gaaR* strain had the strongest reduction in growth on sugar beet pectin, as previously reported (Kowalczyk et al., 2017). However, the deletion of *clrB* resulted in the overall strongest growth defect on sugar beet pulp solid medium, considering the whole composition of this substrate. In contrast, in liquid cultures, the deletion of *araR* had the strongest effect on the exoproteome after both 8 and 24 h, as evidenced by SDS-PAGE analysis. The phenotypic difference between these mutants is most likely related to the different experimental time courses and culturing conditions, which cannot be directly compared (Garrigues et al., 2021).

Moreover, transcriptomic data supported the highest impact on CAZyme production by the deletion of *araR* after 2 h, as Δ*araR* showed a distinct CAZyme pattern compared with that of the reference strain after 8 h. The deletion of *gaaR* resulted in a comparable effect on CAZyme production with that of the Δ*araR* strain after 8 h, showing a higher impact than that of *araR* after 24 h. These results confirm that AraR plays a more important role in the initial degradation of pectin, regulating the release of L-arabinose units from the arabinan and arabinogalactan side chains of pectin, whereas GaaR is more involved in the degradation of the galacturonan backbone (Kowalczyk et al., 2017). Moreover, the deletion of *araR* and *gaaR* indicated a shift toward the utilization of (hemi-)cellulose, evidenced by the increased expression of (hemi-)cellulolytic genes. The upregulation of XlnR-regulated (hemi-)cellulolytic genes has previously been suggested to occur when *gaaR* is deleted (Kowalczyk et al., 2017). After 24 h, the deletion of *clrB* showed the highest impact on CAZyme production, highly reducing the expression of genes encoding CAZymes involved in the degradation of cellulose and xyloglucan.

Our results also show that the deletion of *galX* had only a minor phenotypic impact, evidenced by the further reduced growth of Δ*gaaR*Δ*araR*Δ*rhaR*Δ*galX* compared with the Δ*gaaR*Δ*araR*Δ*rhaR* mutant on L-arabinose. Transcriptome data showed no differential expression of CAZyme or metabolic genes in the Δ*galX* mutant compared with the reference strain at any of the studied timepoints. These results suggest that GalX is not the main TF involved in sugar beet pulp degradation, but contributes to the utilization of L-arabinose and D-galactose (Gruben et al., 2012). ARA1, the functional ortholog of AraR in *Trichoderma reesei*, has been reported to regulate D-galactose catabolism in this fungus (Benocci et al., 2018). However, no correlation between D-galactose responsive regulators and L-arabinose utilization has been reported yet. In *Aspergillus nidulans,* GalX was shown to control GalR, another D-galactose-responsive regulator only present in this species (Christensen et al., 2011), but is not involved in the regulation of pentose catabolism (Kowalczyk et al., 2015). Whether GalX would contribute to L-arabinose metabolism together with AraR in *A. niger* remains to be addressed.

Our results show that the abolished growth on a major component does not indicate the highest impact on the growth on a complex substrate such as sugar beet pulp. Owing to the complexity and redundancy of the regulatory network involved in plant biomass degradation, which is well indicated by the co-regulation of several crucial genes (de Souza et al., 2013; Gruben et al., 2017; Kowalczyk et al., 2017; Kun et al., 2021; Raulo et al., 2016), the analysis of combinatorial deletion strains is required to better understand the regulation of the degradation of specific substrate components.

As expected, the double deletion of *xlnR* and *araR* had a high impact on sugar metabolism, as evidenced by the downregulation of major PCP genes as well as the reduced growth on D-xylose and L-arabinose (Battaglia et al., 2014). The double deletion of *xlnR* and *clrB* resulted in the lowest impact on the expression of CAZyme genes after 8 h, and showed an overall minor reduction in the expression of metabolic genes at all analyzed timepoints. However, both XlnR and ClrB were reported to play an important role in (hemi-)cellulose utilization (Kun et al., 2021; Raulo et al., 2016; van Peij et al., 1998a; Van Peij et al., 1998b), which correlates with the minor phenotypic impact after 8 h, when pectin is the primary carbon source in sugar beet pulp. In contrast, the Δ*xlnR*Δ*clrB* mutant showed a more distinct phenotype after 24 h when the utilization of cellulose was more prominent in the reference strain. The CAZyme gene expression profile of Δ*xlnR*Δ*clrB* mutant indicated that pectin, which was still present in the medium, was the most likely primary carbohydrate utilized by this mutant after 24 h of growth. Moreover, the expression of proteolytic genes was the highest in the Δ*xlnR*Δ*clrB* after 24 h, which supports a reduced ability to grow by utilizing carbohydrates at this timepoint.

The significant increase in expression of the putative pectinolytic genes *abnC*, NRRL3_10498 (putative exo-galactanase), and NRRL3_3855 (putative endo-arabinanase) in most combinatorial deletion mutants indicates that these genes might be part of a back-up system when the expression of major pectinolytic genes is reduced. This hypothesis is also supported by the fact that only the Δ*xlnR*Δ*clrB* double deletion mutant, which still shows expression levels of most major pectinolytic genes comparable with the reference strain, did not show a substantial increase in the expression of these putative back-up genes after 8 h.

Interestingly, all tested strains showed the expression of several genes encoding amylolytic proteins (e.g., *agdB*, *glaA*, *amyA*, *agdA*) in our experiment, despite the absence of starch in sugar beet pulp. These genes are not likely to be directly affected by the studied TFs, and the expression through the amylolytic regulator, AmyR, might be a result of the presence of the released D-glucose in the medium (vanKuyk et al., 2012).

Overall, this study shows that the single and combinatorial deletion of *araR* resulted in a highly altered phenotype, supported by a distinct CAZyme gene expression profile after 2 and 8 h of growth, indicating a shift toward the utilization of alternative carbon sources at the later timepoint. Moreover, the transcriptome data of 8 h samples suggested the additional utilization of proteins in the combinatorial deletion strains carrying the deletion of *araR*, evidenced by the increased expression of proteolytic genes, such as *pepA* or NRRL3_800 (putative tripeptidyl peptidase). The enzyme activity studies also supported a distinct approach for the utilization of sugar beet pulp components for these mutants.

Finally, the Δ*xlnR*Δ*araR*Δ*clrB* and the Δ*gaaR*Δ*araR*Δ*rhaR*Δ*galX*Δ*xlnR*Δ*clrB* mutants showed comparable CAZyme gene expression profiles at all timepoints, as well as comparable proteolytic gene expression profile at the analyzed 8 and 24 h. The data suggest that both of these strains are likely starving after 24 h in the sugar beet pulp liquid cultures. Even though the Δ*xlnR*Δ*araR*Δ*clrB* mutant showed a substantially improved growth compared with the Δ*gaaR*Δ*araR*Δ*rhaR*Δ*galX*Δ*xlnR*Δ*clrB* mutant on solid medium, the overall data indicate that AraR, XlnR, and ClrB are responsible for the regulation of the major activities involved in the efficient degradation and utilization of sugar beet pulp components.

### Limitations of the study

This study shows phenotypic characterization of single and combinatorial deletion mutants of the main TFs involved in sugar beet pulp utilization. Moreover, SDS-PAGE, enzymatic assays, and gene expression profiles of selected strains have been shown, compared, and discussed. Whereas the number of genetic modifications that we can perform in *A. niger* with CRISPR/Cas9 is not a limiting factor, owing to the very high number of strains generated with the combinatorial gene deletion approach, it is not possible to analyze and show all of them in this study. Thus, only a selection of strains was analyzed. The transcriptome data generated in this study show the adaptation of *A. niger* toward the utilization of alternative components of sugar beet pulp when several TFs are deleted. These data also suggest the upregulation of putative pectinolytic genes. Proteomics would also help validate the gene expression data showed in this study through the analysis of the presence of the corresponding proteins in the exoproteome. However, proteomics analyses have not been performed in this study.

## STAR★Methods

### Key resources table

**Table:** 

REAGENT or RESOURCE	SOURCE	IDENTIFIER
**Bacterial and virus strains**		

*Escherichia coli* DH5α	Thermo Fisher Scientific	Cat#EC0112

**Chemicals, peptides, and recombinant proteins**		

4-nitrophenyl α-L-arabinofuranoside	Sigma-Aldrich	Cat#N3641
4-nitrophenyl α-D-galactopyranoside	Sigma-Aldrich	Cat#N0877
4-nitrophenyl β-D-galactopyranoside	Sigma-Aldrich	Cat#N1252
4-nitrophenyl α-L-rhamnopyranoside	Sigma-Aldrich	Cat#N7763
4-nitrophenyl β-D-glucopyranoside	Sigma-Aldrich	Cat#N7006
Azo-galactan (potato)	Megazyme	S-AGALP
Azo-xylan (birchwood)	Megazyme	S-AXBP
Azo-CM-Cellulose	Megazyme	S-ACMC

**Critical commercial assays**		

NucleoSpin RNA, Mini kit for RNA purification	Macherey-Nagel	Cat#740955.250

**Deposited data**		

Transcriptome data	This study	Accession numbers are indicated in the “Data and code availability” section

**Experimental models: Organisms/strains**		

*Aspergillus niger* N593 Δ*kusA* (*cspA1*, *pyrG*^*-*^*, kusA::amdS*); All mutants derived from this strain are listed in Table S3	Westerdijk Fungal Biodiversity Institute culture collection (Utrecht, The Netherlands)	CBS 138852

**Oligonucleotides**		

The complete list of oligonucleotides used in this study is shown in Table S4	Integrated DNA Technologies, Inc. (IDT, Leuven, Belgium)	https://eu.idtdna.com/pages

**Recombinant DNA**		

ANEp8-Cas9-*pyrG* plasmid	(Song et al., 2018)	https://www.addgene.org/117169/

**Software and algorithms**		

Geneious 11.04.4	Biomatters Ltd.	https://www.geneious.com/
STATGRAPHICS Centurion XVI Version 16.1.17	Statgraphics Technologies, Inc.	https://www.statgraphics.com/centurion-xvi
BBDuk	Joint Genome Institute	https://sourceforge.net/projects/bbmap/
HISAT2 version 2.2.0	(Kim et al., 2015)	http://daehwankimlab.github.io/hisat2/download/
deepTools v3.1	(Ramírez et al., 2014)	https://deeptools.readthedocs.io/en/3.1.0/content/tools/computeMatrix.html
FeatureCounts	(Liao et al., 2014)	https://bioinformaticshome.com/tools/rna-seq/descriptions/FeatureCounts.html
R software	R Core Team	https://www.r-project.org/

### Resource availability

#### Lead contact

Further information and requests for resources and reagents should be directed to and will be fulfilled by the lead contact, Ronald P. de Vries (r.devries@wi.knaw.nl).

#### Materials availability

All fungal strains generated in this study were deposited at the culture collection of Westerdijk Fungal Biodiversity Institute (Utrecht, the Netherlands) under the accession numbers listed in Table S3.

### Experimental model and subject details

#### Microbial strains and growth conditions

*Escherichia coli* DH5α was used for plasmid propagation and was grown in Luria-Bertani (LB) medium with 50 μg/mL ampicillin (Sigma-Aldrich) at 37°C.

All fungal strains used in this study (Table S3) are derived from *A. niger* CBS 138852 and were deposited at the culture collection of Westerdijk Fungal Biodiversity Institute (Utrecht, The Netherlands). They were grown and maintained at 30°C on *Aspergillus* Complete Medium (CM) (de Vries et al., 2004) containing 1% D-glucose and supplemented with 1.22 g/L uridine (Sigma-Aldrich). For conidia collection, spores were harvested, dispersed in ACES buffer, and concentration was adjusted using a hemocytometer.

Growth profiles were performed on Minimal Medium (MM) (de Vries et al., 2004) plates containing 25 mM D-glucose, L-arabinose, D-xylose, D-galacturonic acid or L-rhamnose (Sigma-Aldrich); or 1% beechwood xylan, cellulose, xyloglucan, sugar beet pectin or sugar beet pulp. Total sugar composition of the sugar beet pulp used in this study is shown in Table S1. In order to remove residual free sugars, dried and finely ground sugar beet pulp was autoclaved and washed as previously described (Kun et al., 2021). All media were supplemented with 1.22 g/L uridine. Growth profile plates were inoculated in duplicates with 10^3^ conidia and incubated at 30°C for up to 14 days. Growth was monitored daily by visual inspection.

### Method details

#### DNA constructions and fungal transformation

All strains generated in this study were obtained using the CRISPR/Cas9 genome editing system (Song et al., 2018). The autonomous replicative plasmid ANEp8-Cas9-*pyrG* was used in this work. The *A. niger* regulators deleted in this study were AraR (gene ID: NRRL3_07564), XlnR (NRRL3_04034), ClrB (NRRL3_09050), GaaR (NRRL3_08195), RhaR (NRRL3_01496), and GalX (NRRL3_07290). The design of the gRNA sequences (20 bp) was performed using the Geneious 11.04.4 software tool (https://www.geneious.com). The gRNA sequences (Table S4) with no predicted off-targets and the highest on-target activity were designed based on the experimentally determined predictive model described by (Doench et al., 2014). All rescue templates (RTs), which include ∼500 bp of the 5′ and -3′ flanking regions of the target genes, were obtained by fusion-PCR using Phusion High-Fidelity DNA Polymerase (Thermo Fisher Scientific). Two PCR fragments were generated by amplifying ∼600 bp upstream and downstream sequence of the target genes. These two fragments were fused together in a second nested PCR obtaining ∼1,000 bp RT, and were subsequently purified (Wizard® SV Gel and PCR Clean-Up System, Promega).

CRISPR/Cas9 plasmid constructions were performed following protocols described in (Kun et al., 2020; Song et al., 2018), and the generation and transformation of *A. niger* protoplasts were carried out as reported in (Kowalczyk et al., 2017) with some modifications (Kun et al., 2020). One μg ANEp8-Cas9-*pyrG* plasmid, together with 5 μg of each corresponding RT were used for each transformation. Putative mutant strains were purified by two consecutive single colony streaking, followed by cultivation on uridine-containing plates in order to remove the self-replicating plasmid. Candidates carrying the expected mutations were subsequently grown on medium containing 5-fluoro-orotic acid (5-FOA) in order to screen for colonies which have lost the ANEp8-Cas9-*pyrG* plasmid. Single Δ*xlnR*, Δ*araR,* Δ*clrB,* Δ*gaaR,* Δ*rhaR* and Δ*galX* mutants were obtained by single deletion using *A. niger* CBS 138852 as genetic background. The Δ*xlnR*Δ*araR* and Δ*xlnR*Δ*clrB* strains were obtained by simultaneous double deletions using the same parental strain as a background. The triple mutants Δ*xlnR*Δ*araR*Δ*clrB* and Δ*gaaR*Δ*araR*Δ*rhaR* were obtained after a single *araR* deletion in the Δ*xlnR*Δ*clrB*, and Δ*gaaR*Δ*rhaR* genetic backgrounds, respectively. The quadruple Δ*gaaR*Δ*araR*Δ*rhaR*Δ*galX* was obtained after simultaneous *araR* and *galX* deletions in the Δ*gaaR*Δ*rhaR* genetic background. The quintuple Δ*gaaR*Δ*araR*Δ*rhaR*Δ*galX*Δ*xlnR* and Δ*gaaR*Δ*araR*Δ*rhaR*Δ*galX*Δ*clrB* mutant strains were obtained after single *xlnR* or *clrB* deletions in the Δ*gaaR*Δ*araR*Δ*rhaR*Δ*galX* genetic background, respectively. Finally, the sextuple mutant Δ*gaaR*Δ*araR*Δ*rhaR*Δ*galX*Δ*xlnR*Δ*clrB* was obtained after a simultaneous double deletion of *xlnR* and *clrB* in Δ*gaaR*Δ*araR*Δ*rhaR*Δ*galX* genetic background. Mutant strains were confirmed phenotypically (Figure 1) and by analytical PCR through the amplification of each target gene region (data not shown). All primers used in this study are shown in Table S4 and were ordered from Integrated DNA Technologies, Inc. (IDT, Leuven, Belgium).

#### Protein production and enzyme activity assays

Cell free supernatants of 1% washed sugar beet pulp samples from *A. niger* reference strain, Δ*xlnR*, Δ*araR,* Δ*clrB,* Δ*gaaR,* Δ*rhaR,* Δ*galX,* Δ*xlnR*Δ*araR,* Δ*xlnR*Δ*clrB,* Δ*xlnR*Δ*araR*Δ*clrB,* Δ*gaaR*Δ*araR*Δ*rhaR*Δ*galX* and Δ*gaaR*Δ*araR*Δ*rhaR*Δ*galX*Δ*xlnR*Δ*clrB* mutant strains were harvested after 2, 8 and 24 h of growth at 30°C and 250 rpm. Ten μL of each supernatant sample were analyzed by SDS-PAGE using SDS-12% polyacrylamide gels calibrated with PageRuler™ Plus Prestained Protein Ladder (Thermo Scientific), silver stained (Chevallet et al., 2006), and documented using HP Scanjet G2410 scanner. Samples were evaluated in biological duplicates.

Enzyme activities were evaluated by using colorimetric *para*-nitrophenol (*p*NP) or azo-dye substrate assays in 96-well flat bottom microtiter plates. For *p*NP assays, 10 μL 24 h supernatant samples were mixed with 50 μL of 50 mM NaAc (pH 5), 30 μL of demineralized water and 10 μL of 0.1% 4-nitrophenyl α-L-arabinofuranoside (for α -arabinofuranosidase (ABF) activity), 0.1% 4-nitrophenyl α-D-galactopyranoside (for α-galactosidase (AGL) activity), 0.1% 4-nitrophenyl β-D-galactopyranoside (for β-galactosidase (LAC) activity), 0.1% 4-nitrophenyl α-L-rhamnopyranoside (for α-rhamnosidase (RHA) activity) or 0.1% 4-nitrophenyl β-D-glucopyranoside (for β-glucosidase (BGL) activity) in a final volume of 100 μL. The assays were measured after 30–360 min incubation at 30°C. The reactions were stopped by adding 100 μL of 0.25 M Na_2_CO_3_ to the mixture and the absorption values were measured at 405 nm wavelength using a FLUOstar OPTIMA microplate reader (BMG Labtech).

For azo-dye substrate assays, 20 μL 24 h supernatant samples were mixed with 30 μL of 100 mM NaAc (pH 4.6) and 50 μL of Azo-galactan (potato) (Megazyme) (for β-galactanase (GAL) activity), Azo-Xylan (Birchwood) (Megazyme) (for endoxylanase (XLN) activity) or Azo-CM-Cellulose (Megazyme) (for endoglucanase (EGL) activity). The reaction mixtures were incubated for 4 h at 30°C and were terminated by the addition of 250 μL of precipitation solution (4% NaAc∗3H_2_O, 0.4% ZnAc, 76% EtOH, pH 5). The microtiter plates were centrifuged at 4°C, 1000 × g for 10 min. Subsequently, the supernatant samples were transferred to another microtiter plates and the activity was determined based on the absorption measured at 600 nm wavelength using a FLUOstar OPTIMA microplate reader (BMG Labtech).

#### Transcriptomic analysis

For transcriptomic analysis, freshly harvested spores from *A. niger* parental strain, Δ*xlnR*, Δ*araR,* Δ*clrB,* Δ*gaaR,* Δ*rhaR,* Δ*galX,* Δ*xlnR*Δ*araR,* Δ*xlnR*Δ*clrB,* Δ*xlnR*Δ*araR*Δ*clrB,* Δ*gaaR*Δ*araR*Δ*rhaR*Δ*galX* and Δ*gaaR*Δ*araR*Δ*rhaR*Δ*galX*Δ*xlnR*Δ*clrB* mutant strains were pre-grown (10^6^ spores/mL) in 250 mL 2% D-fructose CM supplemented with 1.22 g/L uridine for 16 h at 30°C in a rotary shaker at 250 rpm. After that, mycelia were harvested by filtration through sterile cheesecloth, rinsed with MM, and ∼2.5 g (wet weight) mycelium was transferred into 50 mL MM containing 1% washed sugar beet pulp with 1.22 g/L uridine. Mycelia were collected after 2, 8, and 24 h, frozen in liquid nitrogen, and stored at −80°C until further use. Samples were collected in biological triplicates. The transcriptomes of the parental and mutant strains were analyzed using RNA-seq. RNA was extracted from grinded mycelia using TRIzol reagent (Invitrogen) and purified with NucleoSpin RNA kit for RNA purification (Macherey-Nagel) with DNAse treatment. RNA quality and quantity were assessed by RNA gel electrophoresis and NanoDrop ND-1000 (Thermo Scientific). Purification of mRNA, synthesis of cDNA library and sequencing were conducted at Joint Genome Institute (JGI, California, US). RNA samples were single-end sequenced using Illumina NovaSeq platform (http://illumina.com). Raw fastq file reads were filtered and trimmed using the JGI quality control (QC) pipeline. Using BBDuk (https://sourceforge.net/projects/bbmap/) raw reads were evaluated for artefact sequence by kmer matching (kmer = 25), allowing one mismatch and detected artefact was trimmed from the 3′ end of the reads. RNA spike-in reads, PhiX reads and reads containing any undetermined nucleotides (Ns) were removed. Quality trimming was performed using the phred trimming method set at Q6. Reads under the length threshold were removed (minimum length 25 bases or 1/3 of the original read length - whichever is longer). The cleaned reads were mapped to *A. niger* NRRL3 genome (https://mycocosm.jgi.doe.gov/Aspni_NRRL3_1/Aspni_NRRL3_1.home.html) (Aguilar-Pontes et al., 2018) using HISAT2 version 2.2.0 (Kim et al., 2015). Strand-specific coverage files were generated using deepTools v3.1 (Ramírez et al., 2014). FeatureCounts (Liao et al., 2014) was used to generate the raw gene counts file using gff3 annotations. Only primary hits assigned to the reverse strand were included in the raw gene counts. Raw gene counts were used to evaluate the level of correlation between biological replicates using Pearson’s correlation. Three biological replicates were prepared and sequenced for each condition. Three individual samples were discarded for further analysis due to their poor sequencing quality.

Differentially expressed genes (DEGs) were detected using the R package DESeq2 (Love et al., 2014). Transcripts were considered differentially expressed if the DESeq2 fold change of mutant strains compared to the control was >2 (upregulation) or <0.5 (downregulation) and *p*adj <0.01 and at least one of the two expression values was FPKM >20.

Heat maps for transcriptome data visualization were generated using the “gplots” package of R software, with the default parameters: “Complete-linkage clustering method and Euclidean distance.” The data used for the generation of heat maps is shown in Data S2. Genes with an expression of FPKM <20 in each sample, were excluded from the analysis.

### Quantification and statistical analysis

Enzymatic activity assays were performed by using biological duplicates and technical triplicates. Differences in enzyme activities were determined using the one-way analysis of variance (ANOVA) and Tukey’s honest significant difference (HSD) test (Table S5). Statistical significance was referred for p <0.05. Analyses were done using STATGRAPHICS Centurion XVI Version 16.1.17 (www.statgraphics.com/centurion-xvi).

## Data Availability

Section 1• The RNA-seq data generated in this study have been deposited at the Sequence Read Archive at NCBI (Accession numbers: SRP296371 - SRP296379, SRP296381 - SRP296396, SRP296398 - SRP296424, SRP296426 - SRP296434, SRP296436 - SRP296444, SRP299075 - SRP299083, SRP299091 - SRP299099, SRP308099 - SRP308107, SRP332363 - SRP332366 and SRP332368 - SRP332371). Section 2 • All other data are available in the main text or in the supplemental information files. Section 3 • This paper does not report original code. • Any additional information required to reanalyze the data reported in this paper is available from the lead contact upon request.

## Supplemental information

Document S1. Figures S1–S5 and Tables S1–S5

Data S1. Enzyme activity measurements performed in this study, related to Figure 2

Data S2. Gene expression values of CAZy-, metabolic- and transcription factor genes of *A. niger* reference (CBS 138852) and mutant strains analyzed in this study, related to Figures 3–7
