# Supplementary material for: Unraveling the regulation of sugar beet pulp utilization in the industrially relevant fungus Aspergillus niger
Source: iScience. 2022 Mar 12;25(4):104065. doi: 10.1016/j.isci.2022.104065 (PMC8961234; doi:10.1016/j.isci.2022.104065)
Supplement: Document S1. Figures S1–S5 and Tables S1–S5 [file mmc1.pdf]

iScience, Volume *25*

Supplemental information

Unraveling the regulation of sugar beet pulp utilization in the industrially relevant fungus *Aspergillus niger*

Sandra Garrigues, Roland S. Kun, Mao Peng, Diane Bauer, Keykhosrow Keymanesh, Anna Lipzen, Vivian Ng, Igor V. Grigoriev, and Ronald P. de Vries

Supplementary figures

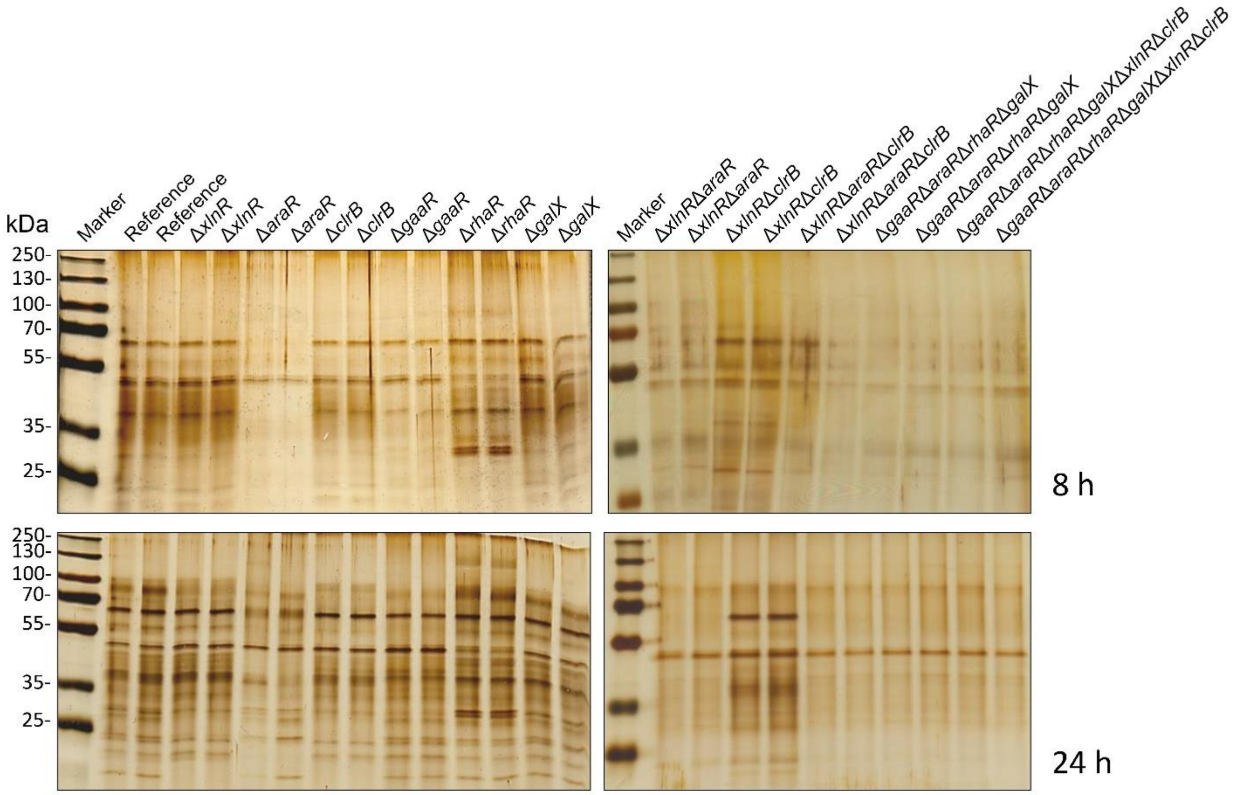

Figure S1. SDS-PAGE analysis of extracellular protein production of *A. niger* reference (CBS 138852) and regulatory mutant strains, related to **Figure 2**. Samples originated from 8, and 24 h growth in 1% liquid sugar beet pulp and were evaluated in biological duplicates.

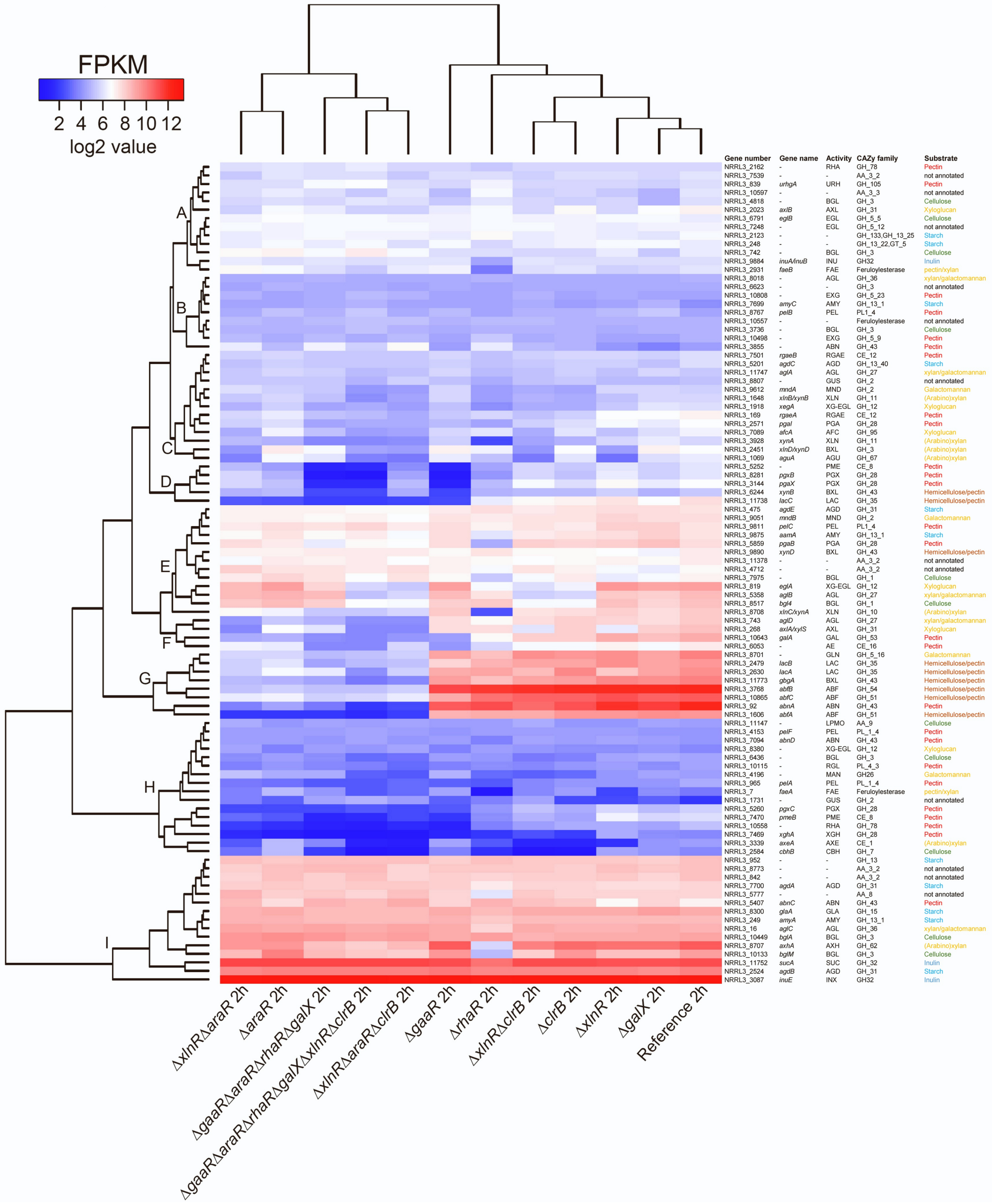

Figure S2. Hierarchical clustering of CAZy-encoding genes in *A. niger* reference (CBS 138852) and deletion mutant strains, related to **Figure 7**. Gene expression data originated from 2 h of growth in 1% sugar beet pulp liquid cultures. The substrates associated with the corresponding genes are indicated by different colors. Enzyme activity abbreviations are described in **Table S2**.

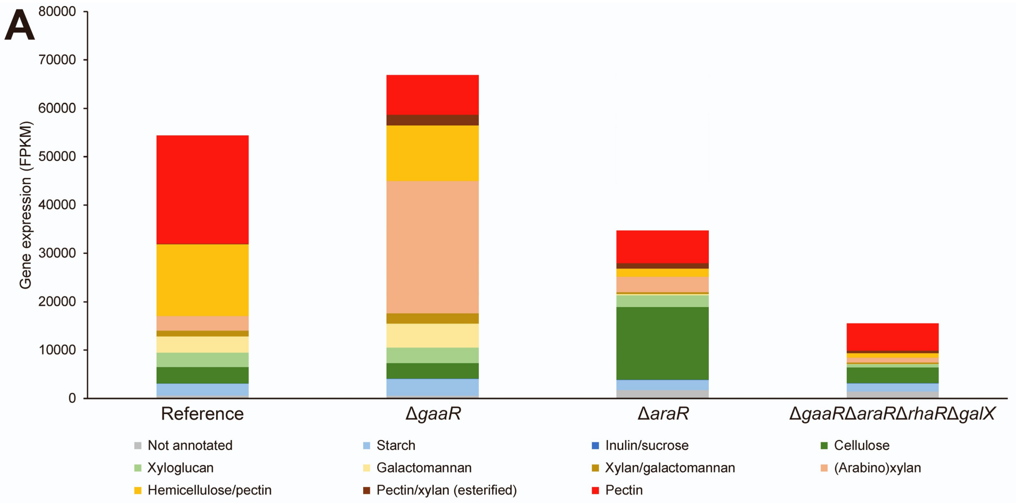

B

Substrate	Reference	Δ*gaaR*	Δ*araR*	Δ*gaaR* Δ*araR* Δ*rhaR* Δ*galX*
Pectin	34	17	23	10
Pectin/xylan (esterified)	2	2	2	2
Hemicellulose/pectin	11	10	10	7
(Arabino)xylan	7	8	7	6
Xylan/galactomannan	5	5	4	4
Galactomannan	3	3	2	2
Xyloglucan	8	8	7	4
Cellulose	13	18	19	14
Inulin/sucrose	1	1	2	2
Starch	10	11	12	12
Not annotated	8	7	12	10

Number of genes

Low High

Figure S3. Expression of CAZy-encoding genes in the reference (CBS 138852) and Δ*araR*, Δ*gaaR* and Δ*gaaR*Δ*araR*Δ*rhaR*Δ*galX* deletion strains, related to **Figure 7**. (A) Cumulative expression of genes associated with the degradation of specific substrates after 8 h of growth in 1% sugar beet pulp liquid cultures. (B) Number of genes associated with the degradation of each substrate after 8 h of growth. Only genes with an expression value of FPKM > 20 were considered for this analysis.

A

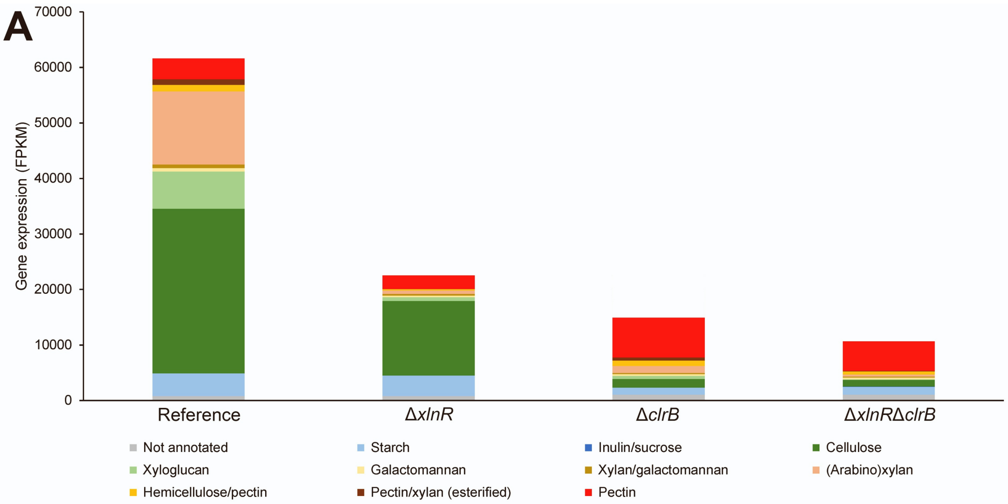

B

Substrate	Reference	Δ*xlnR*	Δ*clrB*	Δ*xlnR*Δ*clrB*
Pectin	30	22	28	27
Pectin/xylan (esterified)	3	0	1	1
Hemicellulose/pectin	9	5	9	5
(Arabino)xylan	8	6	6	3
Xylan/galactomannan	4	3	3	4
Galactomannan	4	4	2	2
Xyloglucan	8	5	5	2
Cellulose	19	15	11	10
Inulin/sucrose	0	0	0	1
Starch	12	11	10	11
Not annotated	9	9	11	12

Number of genes

Low High

Figure S4. Expression of CAZy-encoding genes in the reference (CBS 138852) and Δ*clrB*, Δ*xlnR* and Δ*xlnR*Δ*clrB* deletion strains, related to **Figure 7**. (A) Cumulative expression of genes associated with the degradation of specific substrates after 24 h of growth in 1% sugar beet pulp liquid cultures. (B) Number of genes associated with the degradation of each substrate after 24 h of growth. Only genes with an expression value of FPKM > 20 were considered for this analysis.

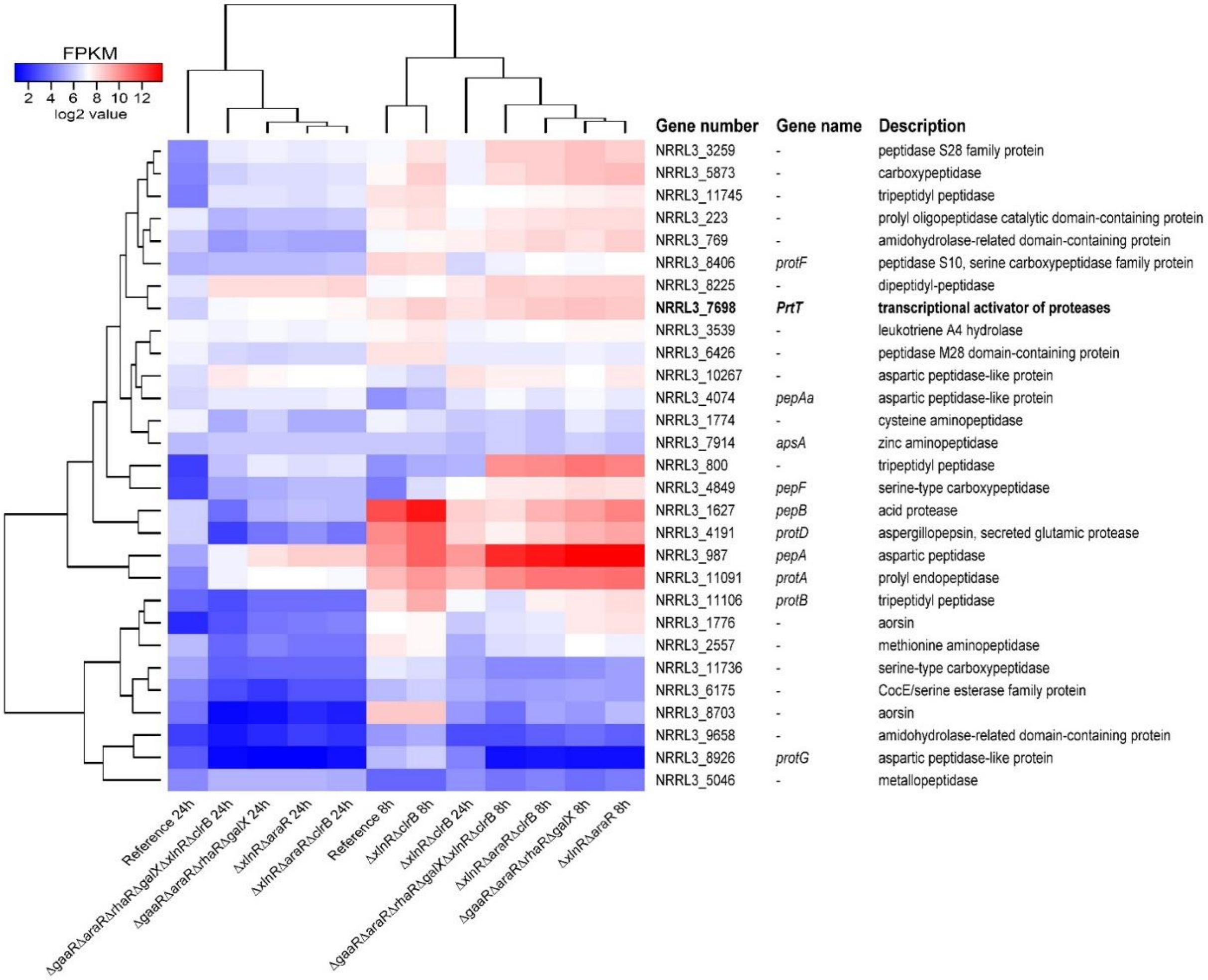

Figure S5. Hierarchical clustering of genes involved in proteolytic activities in *A. niger* reference (CBS 138852) and combinatorial deletion mutant strains, related to **Figure 7**. The *prtT* gene encoding the proteolytic transcription factor PrtT is highlighted in bold. Gene expression data originated from 8 h and 24 h of growth in 1% sugar beet pulp liquid cultures.

Supplementary tables

Table S1. Sugar composition of sugar beet pulp used in this study, related to STAR Methods. Composition analysis was performed as previously described (Mäkelä et al., 2017).

mol%							w/w%
L-arabinose	D-xylose	D-mannose	D-galactose	D-galacturonic acid	L-rhamnose	D-glucose	Total sugar
29	2.3	2.1	6.5	27	1.4	32	56

Table S2. Abbreviations of enzyme activities presented in this study, related to **Figure 5** and **Figure 7**.

Abbreviation	Enzyme activity
ABF	α-L-arabinofuranosidase
ABN	endo-arabinanase
AE	acetyl esterase
AFC	α-L-fucosidase
AGD	α-glucosidase
AGL	α-1,4-galactosidase
AGU	α-glucuronidase
AMY	α-amylase
AXE	acetylxylan esterase
AXH	arabinoxylan arabinofuranohydrolase
AXL	α-xylosidase
BGL	β-1,4-glucosidase
BXL	β-1,4-xylosidase
CBH	cellobiohydrolase
EGL	β-1,4-endo-glucanase
EXG	exo-1,3-galactanase
FAE	feruloyl esterase
GAL	β-1,4-endo-galactanase
GLA	glucoamylase
GLN	exo-1,6-galactanase
GUS	β-glucuronidase
INU	endo-inulinase
INX	exo-inulinase
LAC	β-1,4-galactosidase
LPMO	lytic polysaccharide monooxygenase
MAN	β-1,4-endo-mannanase
ML-EGL	β-1,3/β-1,4-endoglucanase
MND	β-1,4-mannosidase
PEL	pectin lyase
PGA	endo-polygalacturonase
PGX	exo-polygalacturonase
PLY	pectate lyase
PME	pectin methyl esterase
RGAE	rhamnogalacturonan acetyl esterase
RGL	rhamnogalacturonan lyase
RGX	exo-rhamnogalacturonase
RHA	α-rhamnosidase
RHG	endo-rhamnogalacturonase
SUC	invertase/β-fructofuranosidase
URH	unsaturated rhamnogalacturonyl hydrolase
XG-EGL	xyloglucanase
XGH	xylogalacturonase
XLN	β-1,4-endo-xylanase

Table S3. *A. niger* strains used in this study, related to STAR Methods.

CBS number	Strain description	Genotype	Reference
CBS 138852	N593 Δ*kusA*	*cspA1*, *pyrG*$^-$, *kusA::amdS*	(Meyer et al., 2007)
CBS 145451	Δ*araR*	*cspA1*, *pyrG*$^-$, *kusA::amdS*, Δ*araR*	(Kun et al., 2021)
CBS 145447	Δ*xlnR*	*cspA1*, *pyrG*$^-$, *kusA::amdS*, Δ*xlnR*	(Kun et al., 2021)
CBS 146899	Δ*rhaR*	*cspA1*, *pyrG*$^-$, *kusA::amdS*, Δ*rhaR*	This study
CBS 146900	Δ*galX*	*cspA1*, *pyrG*$^-$, *kusA::amdS*, Δ*galX*	This study
CBS 146901	Δ*gaaR*	*cspA1*, *pyrG*$^-$, *kusA::amdS*, Δ*gaaR*	This study
CBS 145445	Δ*clrB*	*cspA1*, *pyrG*$^-$, *kusA::amdS*, Δ*clrB*	(Kun et al., 2021)
CBS 145455	Δ*xlnR*Δ*araR*	*cspA1*, *pyrG*$^-$, *kusA::amdS*, Δ*xlnR*, Δ*araR*	(Kun et al., 2021)
CBS 145449	Δ*xlnR*Δ*clrB*	*cspA1*, *pyrG*$^-$, *kusA::amdS*, Δ*xlnR*, Δ*clrB*	(Kun et al., 2021)
CBS 145457	Δ*xlnR*Δ*araR*Δ*clrB*	*cspA1*, *pyrG*$^-$, *kusA::amdS*, Δ*xlnR*, Δ*araR*, Δ*clrB*	(Kun et al., 2021)
CBS 146906	Δ*gaaR*Δ*araR*Δ*rhaR*	*cspA1*, *pyrG*$^-$, *kusA::amdS*, Δ*gaaR*, Δ*araR*, Δ*rhaR*	This study
CBS 146907	Δ*gaaR*Δ*araR*Δ*rhaR*Δ*galX*	*cspA1*, *pyrG*$^-$, *kusA::amdS*, Δ*gaaR*, Δ*araR*, Δ*rhaR*, Δ*galX*	This study
CBS 146908	Δ*gaaR*Δ*araR*Δ*rhaR*Δ*galX*Δ*xlnR*	*cspA1*, *pyrG*$^-$, *kusA::amdS*, Δ*gaaR*, Δ*araR*, Δ*rhaR*, Δ*galX*, Δ*xlnR*	This study
CBS 146909	Δ*gaaR*Δ*araR*Δ*rhaR*Δ*galX*Δ*clrB*	*cspA1*, *pyrG*$^-$, *kusA::amdS*, Δ*gaaR*, Δ*araR*, Δ*rhaR*, Δ*galX*, Δ*clrB*	This study
CBS 146910	Δ*gaaR*Δ*araR*Δ*rhaR*Δ*galX*Δ*xlnR*Δ*clrB*	*cspA1*, *pyrG*$^-$, *kusA::amdS*, Δ*gaaR*, Δ*araR*, Δ*rhaR*, Δ*galX*, Δ*xlnR*, Δ*clrB*	This study

Table S4. Primers used in this study, related to STAR Methods. Homology flanks are highlighted in red.

Primer ID	Sequence (5'- 3')	Description
**Primers for construction of repair templates**		
araR-5F-new	GTCCGCAAGTTGTGTGGTGG	Amplification of 5' flanking region of *araR*
araR-5R-new	GCATCGGTGCTGTGAGAAACGGAATCGCAGTCTGATGAAACG	
araR-3F-new	CGTTTCATCAGACTGCGATTCCGTTTCTCACAGCACCGATGC	Amplification of 3' flanking region of *araR*
araR-3R-new	AACCGAGAAGCCCAGTTTCG	
araR-NEST-F-new	GAAGCGACCTCATAGCGACC	5' + 3' flank fusion of *araR* repair template
araR-NEST-R-new	ATGCCAGAAACATGCGATGC	
gaaR-5F	AAAGCAATCACGGCAACTGG	Amplification of 5' flanking region of gaa*R*
gaaR-5R	GGGTATCATCGCTCTAGTATTCACTGATGTGGGAAGCGAATAGAGG	
gaaR-3F	CCTCTATTCGCTTCCCACATCAGTGAATACTAGAGCGATGATACCC	Amplification of 3' flanking region of gaa*R*
gaaR-3R	TGTAGAATACTTGCCCATAGGTCG	
gaaR-NEST-F	AGAATAGCTTCTCTTGCTAGACTGG	5' + 3' flank fusion of *gaaR* repair template
gaaR-NEST-R	CGTGGACGCATTCATCTGTATG	
rhaR-5F	GTGCGGATCTCTTTGATGCG	Amplification of 5' flanking region of gaa*R*
rhaR-5R	GCGATAAACTGCCTCAAAGATGTTTTATCGGGAGATAGACAGGTGC	
rhaR-3F	GCACCTGTCTATCTCCCGATAAAACATCTTTGAGGCAGTTTATCGC	Amplification of 3' flanking region of gaa*R*
rhaR-3R	CATCTTGACTTAGTGAACAGGAGC	
rhaR-NEST-F	CAGTAGTTTAGCGGAGTGGG	5' + 3' flank fusion of *gaaR* repair template
rhaR-NEST-R	ACTCACCTCTTCTCTCATTCTCG	
galX-5F	CGTAGTTGAATGCGACCTGC	Amplification of 5' flanking region of *galX*
galX-5R	CCTTTCAGAGCTTTGAGATGGAAGAGGGAAGAATGAGAAGTCAACCG	
galX-3F	CGGTTGACTTCTCATTCTTCCCTCTTCCATCTCAAAGCTCTGAAAGG	Amplification of 3' flanking region of *galX*
galX-3R	AACAATTCAATTCGCAGAAGCAGC	
galX-NEST-F	GCTCAAACTGCGGACAATGC	5' + 3' flank fusion of *galX* repair template
galX-NEST-R	CCAGTGCGATCAGAAACACG	
XlnR-5F-new	GTGTGTGTGTGAGAGAGAAAGG	Amplification of 5' flanking region of *xlnR*
XlnR-5R-new	GCATCTCATCATCAGCCGTGTGGAAAGTGAGGTATTCAGACCG	
XlnR-3F-new	CGGTCTGAATACCTCACTTTCCACACGGCTGATGATGAGATGC	Amplification of 3' flanking region of *xlnR*
XlnR-3R-new	GACGAGAGGAGTTGGTAGCG	
XlnR-NEST-F	CTTTCTCGTGGGTTCTTCACC	5' + 3' flank fusion of *xlnR* repair template
XlnR-NEST-R-new	GGATGTAGTCGTCCAGGAGG	
clrB-5F	ATCACACAACCCTTCTCGTACC	Amplification of 5' flank region of *clrB*
clrB-5R	CGATAGCGAATCCTAGCAGTTGCTGGAGTTTTTGTTTGACGG	
clrB-3F	ACTGCTAGGATTCGCTATCGCTGTACTTACAGGGTGCAGC	Amplification of 3' flank region of *clrB*
clrB-3R	GGATGGATCGTCTTAGGATGC	
clrB-NEST-F	ATCACACAACCCTTCTCGTACC	5' + 3' flank fusion of *clrB* repair template
clrB-NEST-R	GGATGGATCGTCTTAGGATGC	

**Primers for CRISPR/Cas9 sgRNA construction**		
P1-gRNA	CAACCTCCAATCCAATTTGACTCCGCCGAACGTACTG	5F-sgRNA; for amplification of 5' flank region and fusion of sgRNA construct
P2-gRNA	ACTACTCTACCACTATTTGAAAAGCAAAAAAGGAAGGTACAAAAAAGC	3R-sgRNA; for amplification of 3' flank region and fusion of sgRNA construct
P3-*araR*	CCCAGAAAGTCAGGGCACACGACGAGCTTACTCGTTTCG	5R-*araR*; for amplification of 5' flank region of sgRNA construct
P4-*araR*	GTGTGCCCTGACTTTCTGGGGTTTTAGAGCTAGAAATAGCAAG	3F-*araR*; for amplification of 3' flank region of sgRNA construct
P3-*gaaR*	CGTCGCGTCCATGCCGAACCGACGAGCTTACTCGTTTCG	5F-*gaaR*; for amplification of 5' flank region and fusion of sgRNA construct
P4-*gaaR*	GGTTCGGCATGGACGCGACGGTTTTAGAGCTAGAAATAGCAAG	3R-*gaaR*; for amplification of 3' flank region and fusion of sgRNA construct
P3-*rhaR*	CGCTGTGATCCCGTTCCAGAGACGAGCTTACTCGTTTCG	5F-*rhaR*; for amplification of 5' flank region and fusion of sgRNA construct
P4-*rhaR*	TCTGGAACGGGATCACAGCGGTTTTAGAGCTAGAAATAGCAAG	3R-*rhaR*; for amplification of 3' flank region and fusion of sgRNA construct
P3-*galX*	ATAGTGTCCACCAAACGCGTGTTTTAGAGCTAGAAATAGCAAG	5F-*galX*; for amplification of 5' flank region and fusion of sgRNA construct
P4-*galX*	ACGCGTTTGGTGGACACTATGACGAGCTTACTCGTTTCG	3R-*galX*; for amplification of 3' flank region and fusion of sgRNA construct
P3-*xlnR*	CGGTCTCCTGGCGAGTATGCGACGAGCTTACTCGTTTCG	5R-*xlnR*; for amplification of 5' flank region of sgRNA construct
P4-*xlnR*	GCATACTCGCCAGGAGACCGGTTTTAGAGCTAGAAATAGCAAG	3F-*xlnR*; for amplification of 3' flank region of sgRNA construct
P3-*clrB*	TCCTTTCTTCAGACCTGAGCGACGAGCTTACTCGTTTCG	5R-*clrB*; for amplification of 5' flank region of sgRNA construct
P4-*clrB*	GCTCAGGTCTGAAGAAAGGAGTTTTAGAGCTAGAAATAGCAAG	3F-*clrB*; for amplification of 3' flank region of sgRNA construct
**Primers for screening transformant colonies**		
araR-5F-new	GTCCGCAAGTTGTGTGGTGG	for screening the presence/absence of *araR* gene
araR-3R-new	AACCGAGAAGCCCAGTTTCG	
gaaR-5F	AAAGCAATCACGGCAACTGG	for screening the presence/absence of *gaaR* gene
gaaR-3R	TGTAGAATACTTGCCCATAGGTCG	
rhaR-5F	GTGCGGATCTCTTTGATGCG	for screening the presence/absence of *rhaR* gene
rhaR-3R	CATCTTGACTTAGTGAACAGGAGC	
galX-NEST-F	GCTCAAACTGCGGACAATGC	for screening the presence/absence of *galX* gene
galX-3R	AACAATTCAATTCGCAGAAGCAGC	
XlnR-5F-new	GTGTGTGTGTGAGAGAGAAAGG	for screening the presence/absence of *xlnR* gene
XlnR-3R-new	GACGAGAGGAGTTGGTAGCG	
clrB-F	GTGTGACTGTCCCCTTCTTCC	for screening the presence/absence of *clrB* gene
clrB-R	TTCCTTATGTAGCCCCAGAGC	

Table S5. Summary of the ANOVA analysis for each enzymatic assay, related to STAR Methods and **Figure 2**.

**Condition**	**N. of variables**[a]	**DF**[b]	**F-value**	***p*-value**[c]
ABF	12	11	1133.42	0.0000
AGL	12	11	522.54	0.0000
BGL	12	11	563.29	0.0000
EGL	12	11	33.24	0.0000
GAL	12	11	271.61	0.0000
RHA	12	11	263.9	0.0000
XLN	12	11	115.13	0.0000
LAC	12	11	158.81	0.0000

[a] Number of variables within each ANOVA analysis

[b] DF: Degrees of freedom

[c] *p*-values of the F-test. Statistical significance is referred when $p < 0.05$
